# Multiple gene substitution by Target-AID base-editing technology in tomato

**DOI:** 10.1038/s41598-020-77379-2

**Published:** 2020-11-24

**Authors:** Johan Hunziker, Keiji Nishida, Akihiko Kondo, Sanae Kishimoto, Tohru Ariizumi, Hiroshi Ezura

**Affiliations:** 1 Graduate School of Life and Environmental Sciences, University of Tsukuba, Tsukuba, Japan; 2 Graduate School of Science, Technology and Innovation, Kobe University, Kobe, Japan; 3 Institute of Vegetable and Floricultural Science, NARO, Tsukuba, Ibaraki Japan; 4 Faculty of Life and Environmental Sciences, University of Tsukuba, Tsukuba, Japan; 5 Tsukuba Plant Innovation Research Center, University of Tsukuba, Tsukuba, Japan

**Keywords:** Biotechnology, Plant sciences

## Abstract

The use of Target activation-induced cytidine deaminase (Target-AID) base-editing technology with the CRISPR-Cas 9 system fused with activation-induced cytidine deaminase (AID) resulted in the substitution of a cytidine with a thymine. In previous experiments focusing on a single target gene, this system has been reported to work in several plant species, including tomato (*Solanum lycopersicum* L.). In this research, we used Target-AID technology to target multiple genes related to carotenoid accumulation in tomato. We selected 3 genes, *SlDDB1*, *SlDET1* and *SlCYC-B,* for their roles in carotenoid accumulation. Among 12 edited T_0_ lines, we obtained 10 independent T_0_ lines carrying nucleotide substitutions in the three targeted genes, with several allelic versions for each targeted gene. The two edited lines showed significant differences in carotenoid accumulation. These results demonstrate that Target-AID technology is a highly efficient tool for targeting multiple genes with several allelic versions.

## Introduction

Gene editing is a technology based on the capacity to modify a target gene by removing, inserting or substituting DNA. Base-editing (BE) technology has been developed with the aim of inducing local targeted nucleotide substitutions. The clustered regularly interspaced short palindromic repeats (CRISPR)/CRISPR-associated protein 9 (CRISPR/Cas9) system is the gene editing tool that currently appears to be the most popular tool due to its efficiency and application to any living organism^1^. CRISPR/Cas9 acts as molecular scissors with two nuclease domains inducing DNA double-strand breaks (DSBs). A DSB can be fixed by a nonhomologous end joining (NHEJ) during mitosis after the duplication of DNA. Additionally, in the presence of a homologous DNA template containing the desired DNA sequence, such cleavage can be fixed through homology-directed repair (HDR) during the resorption of DSBs, which involves the introduction of the desired sequence through homologous recombination. Both processes result in the formation of abundant indels, rather than substitutions alone, inducing gene knockout, which can be deleterious depending on the gene or the position within it. To improve the efficiency of substitution, a number of BE technologies have been developed.

Some major BE technologies are based on an engineered Cas9 protein fused with a single strand DNA-specific cytidine deaminase (CDA)^2–4^. Another BE technique has been developed based adenosine deaminase (ADA)^5^ but has only been tested in bacteria. Recently, the CDA and ADA technologies were fused in a single plasmid to form a protein complex that is able to specifically modify nucleotides^6^. Such technologies have been studied in organisms such as bacteria^3,6^, animals^7–9^ and plants^5,10–12^, with a variable range of efficiency. Some of these technologies have been reviewed but are still being improved^13^. Target-AID technology, in which an *Arabidopsis thaliana*-optimized codon nickase Cas9 (nCas9) fused with a lamprey CDA is used, has been demonstrated as a powerful tool for precise BE in bacteria for multiplexing applications^14^. However, in plants, only single gene targets in rice and tomato have been reported thus far^12^.

Tomato is one of the most important commercial horticulture crops grown in the world. The improvement of yield and quality in tomato is a major challenge for breeders, as fruit yield and quality are controlled by several genes acting either independently or in concert. The identification of genes and alleles and their rapid integration in elite cultivars are necessary but time-consuming and expensive processes, as the insertion of a new allelic version in an elite line requires several years and crossing between generations, as observed for the introgression of mutations isolated among mutant collections of the Micro-Tom cultivar^15^. The recent development of gene editing technologies using programmable nucleases appears to be a promising and efficient approach for both breeding and basic research involving forward and reverse genetics in tomato.

Tomato fruit is known as a good source of lycopene, vitamin C, β-carotene, folate, and potassium^16^. Carotenoids play an important role in human nutrition because of their pro-vitamin A activity. However, there are other significant health benefits that are attributed to carotenoids, which are thought to be associated with their activity as antioxidants, such as their anticancer potential. Several genes involved in the improvement of carotenoid accumulation have been identified in tomato during the last two decades, such as tomato DNA Damage UV Binding protein 1 (*SlDDB1*)^17,18^, deetiolated1 (*SlDET1*)^19^ and Lycopene beta cyclase (*SlCYC-B*)^20^ for lycopene accumulation or with *SlCYC-B*^20–22^ and *SlCrtISO*^23^ for β-carotene accumulation. *Slhp1* and *Slhp2* are mutations corresponding to *SlDDB1* and *SlDET1* genes respectively. These mutants confer the unique characteristics including purple roots resulting from anthocyanin accumulation^19,24^, dwarfism, dark leaves and dark green fruits^17–19^.

In this study, we tested Target-AID technology for generation of substitution mutations in multiple genes. As a model experiment, we targeted three genes, *SlDDB1*, *SlDET1* and *SlCYC-B*, which are responsible for carotenoid accumulation, especially that of lycopene in tomato. For this purpose, we used a multiplexing target plasmid carrying three sgRNAs targeting the *SlDDB1*, *SlDET1* and *SlCYC-B* genes with an nCas9-CDA-UGI (Uracil-DNA glycosylase inhibitor) protein complex. This approach resulted in the generation of several lines showing substitutions in each target gene simultaneously, demonstrating the usefulness of the Target-AID technology for multiple gene substitutions in tomato.

## Results

### Target site selection in *SlDDB1, SlDET1 *and *SlCYC-B*

**Figure 1:**
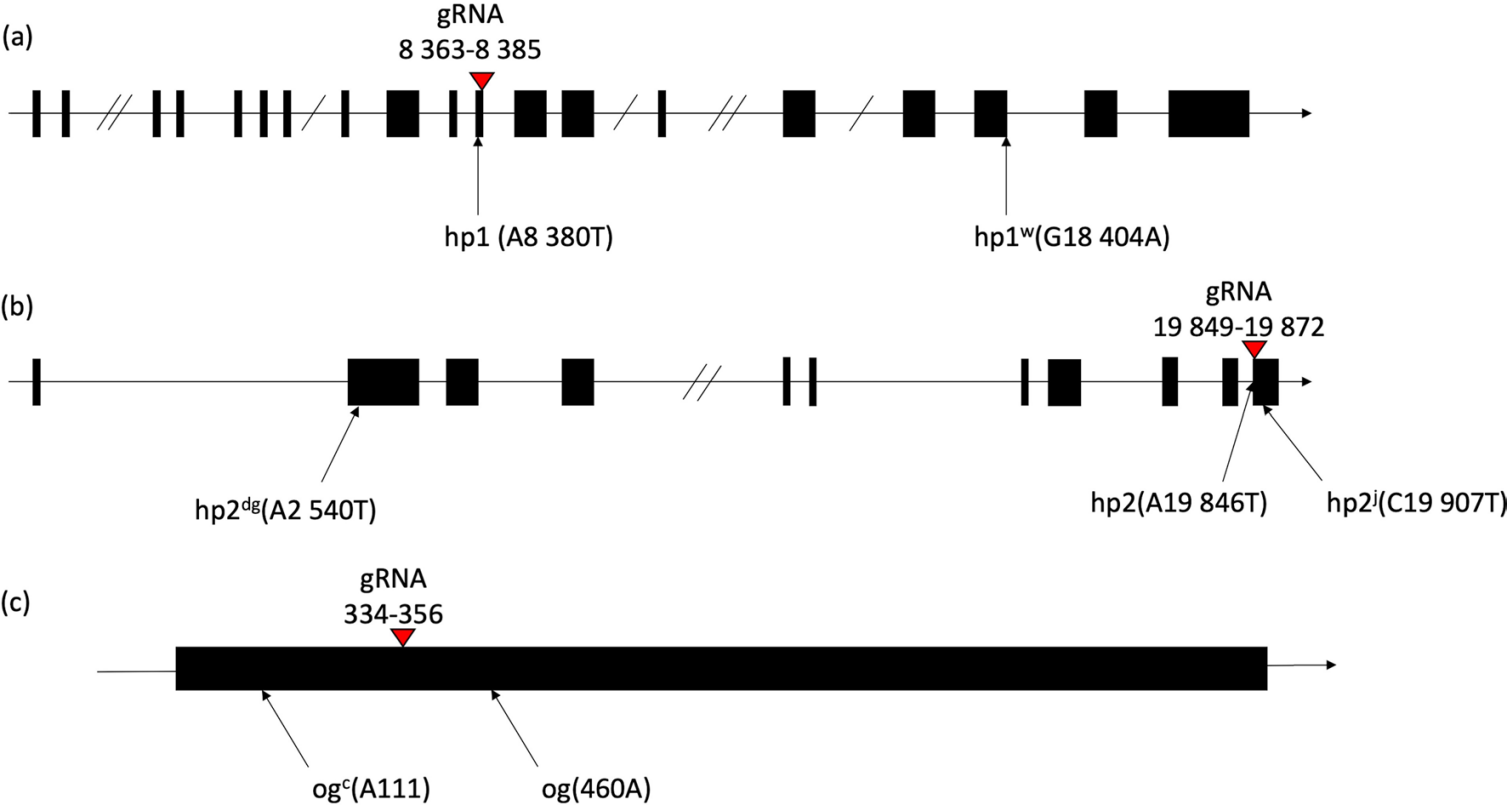
Representation of SlDDB1, SlDET1 and SlCYC-B target sites. Schematic representation of each candidate gene, (**a**) *SlDDB1*, (**b**) *SlDET1* and (**c**) *SlCYC-B*, with the previously described mutations and their corresponding annotations with mutation position compared to the first nucleotide of start codon on gene sequence. Black boxes represent exons regions and lines represent introns or UTR regions. Double slashes in introns represent reduction of the intron size. The red triangles represent the site targeted by the Target-AID with their corresponding nucleotides localization on gene.

As reported previously in tomato, individual mutations in *SlDDB1*^17,18^, *SlDET1*^19^ and *SlCYC-B*^20^, particularly the *Slhp1*, *Slhp2* and *Slog* mutations*,* respectively, result in lines presenting high accumulation of lycopene. Regions close to each original mutation were selected as target site to obtain a new allelic version of each gene (Fig. 1). For *SlDDB1*, the original *Slhp1* mutation is located at nucleotide 8380, where a guanine is substituted by an adenine, resulting in the substitution of an Asp by a Tyr. Because an sgRNA containing this guanine could not be designed, the nucleotide targeted in our construct by using a reverse sgRNA was the guanine located at nucleotide 8383, resulting in the expected substitution of Ala by Pro. For *SlDET1*, the original *Slhp2* mutation, consisting of an AG-to-TG substitution, is located at the junction of intron 10 and exon 11 inducing alternative splicing of the mRNA leading to the deletion of 3 amino acids (Gly, Pro and Glu) of exon 11 within the second Nuclear localization signal (NLS). As the construct could not reproduce the exact substitution, the target region in the exon is expected to produce a nonviable NLS domain, inducing a loss of function resulting in an *hp* phenotype. *For SlCYC-*B, the reported mutations involve the deletion of an adenine at nucleotide 111 for the *Slog*^*c*^ allele and an insertion around nucleotide 460 for the *Slog* allele, resulting in a frameshift of the protein in both cases and the production of a nonfunctional enzyme. An expected target site presenting the possibility of introducing a stop codon by cytidine substitution has been selected, resulting in a truncated protein. Based on these assumptions, we expected that a construct inducing only a substitution instead of an indel could produce nonlethal new allelic versions of the gene, with a potential combination of mutated *SlDDB1* and *SlDET1*, inducing a complementary improvement of lycopene accumulation, as synergistic phenotype could be observed on hypocotyl phenotype^18^. All targets are summarized in Table 1 and schematically represented within the genes in Fig. 1.

**Table 1:**
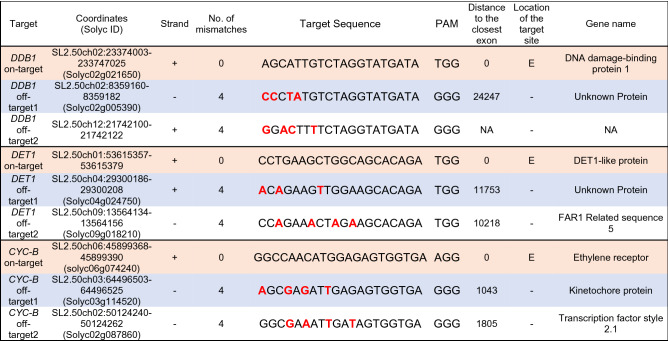
Prediction of *SlDDB1*, *SlDET1* and *SlCYC-B* off-target sites.

Distance to the closest exon: 0 indicates that the target site and exon coordinates overlap; NA indicates that the target sites are farther than 100 kb from the next exon. Location of the target site: E, - indicate exonic, intronic and intergenic, respectively.

### Efficiency of base-editing at the targeted site in the T_0_ lines

**Figure 2:**
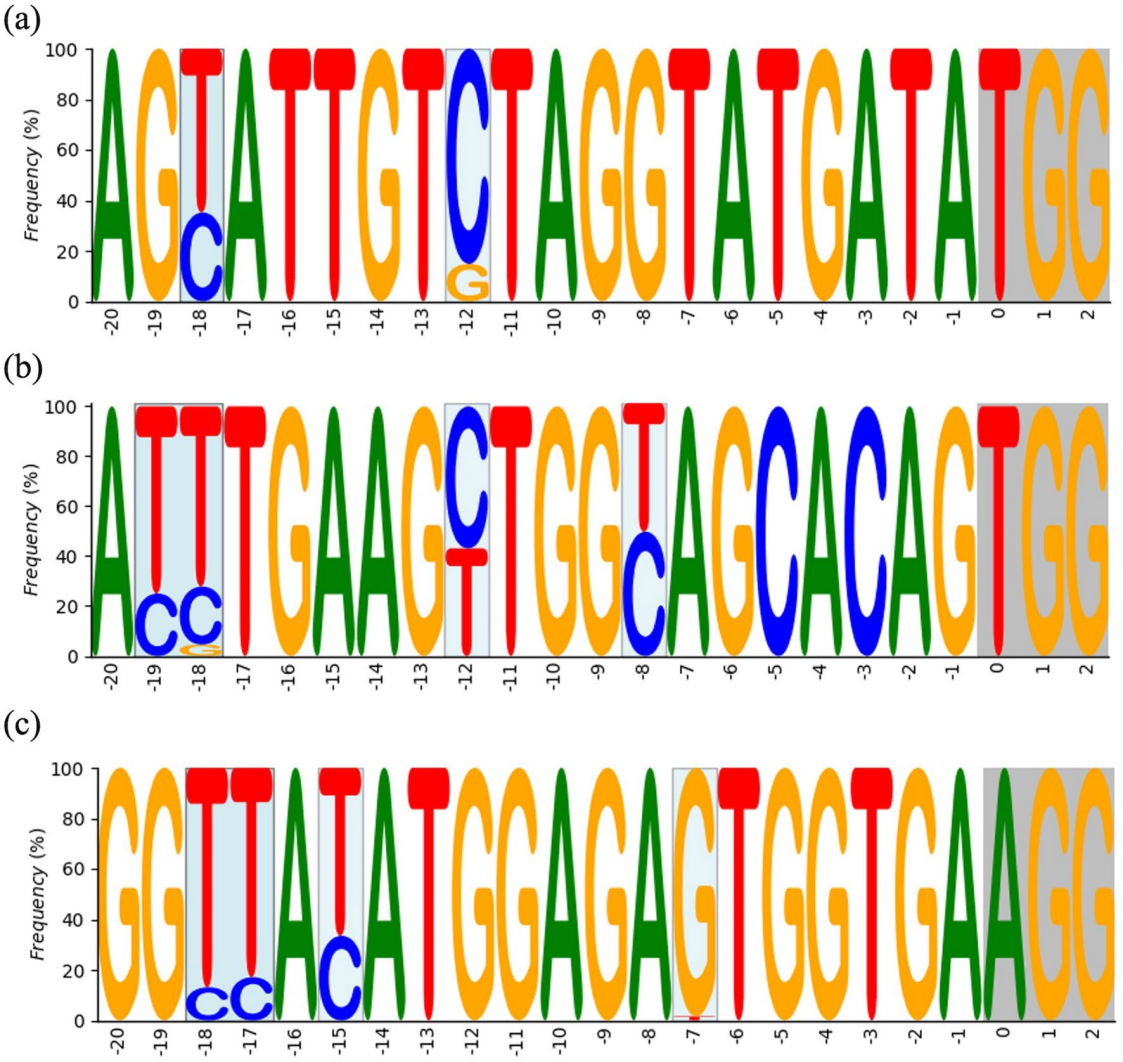
Logo Fig. of the deep sequencing analysis of on-target sites following Target-AID induction in tomato. Representation of the pattern of mutation induced by Target-AID with the corresponding sgRNA sequence in (**a**) Sl*DDB1*, (**b**) Sl*DET1* and (**c**) Sl*CYC-B*. Light blue background represents substitution areas, grey background represents PAM sequences. Numbers under the sequence represent the position of nucleotide from the PAM sequence, zero starting from the nucleotide N of NGG PAM sequence.

Approximately 13 T_0_ lines from 9 independent calli resulting from 151 explants infected were regenerated. It represents about 8% of diploid plants that were transformed via the infection of cotyledons. One regenerated line was identified as a positive control because it did not contain the selection marker and was excluded from further analysis, remaining 12 plants as transgenic. The remaining 12 T_0_ plants (#8, #16, #16-1, #16-2, #16-3 ,#18, #26, #27, #30, #36-2, #43-1, #43-2) showed a typical *hp* mutant phenotype involving dwarfism, dark leaves and dark green fruits, observable in segregating generation (data not show). The sequencing of each target gene was performed to confirm the presence of a substitution rather of an indel. Results show 11 lines among 12 lines showed simultaneous substitution on 3 targets at the same time (Table 2a), resulting in a ratio of about 91.6% of efficiency of edition. Seven regenerated lines were isolated (#16, #16-1, #16-2, #16-3, #18, #43-1, #43-2), which provided the T_1_ seeds. As plants in the T_0_ generation show heterozygotic or chimeric status according to Sanger sequencing, these lines were used to perform amplicon sequencing by NGS to confirm the base-editing efficiency of our construct. Most of the lines showed a high efficiency of cytidine to thymine substitution at the 3′ site of the gRNA for all 3 target genes, with the same pattern of mutation being observed in all independent lines (Fig. 2, Supplementary Tables S1a, S2). Regarding off-target effects, none of the lines presented any significant mutation obtained from the construct, as no substitutions could be observed among the few identified PCR errors (Supplementary Table S1b). Interestingly, no indels for *SlDET1* were present in the T_0_ lines, whereas for *SlDDB1* and *SlCYC-B*, the proportion of total reads showing indels was highly variable, ranging from approximately 12% to more than 90% of the total number of reads (Supplementary Table S1a). The NGS data and the T_0_ Sanger data are summarized in Supplementary Tables S1, S2, respectively. All of these lines were generated to segregate the mutation pattern in the T_1_ generation.

**Table 2:** Mutation type obtained in T_0_ and T_1_ generations of edited tomato lines.

Plant lines	*SlDDB1*		*SlDET1*		*SlCYC-B*	
	Indel	substitution	Indel	substitution	Indel	substitution
**(a)**						
#8	+	+	−	+	−	+
#16	+	+	−	+	−	+
#16-1	+	+	−	+	+	+
#16-2	+	+	−	+	–	+
#16-3	+	+	−	+	+	+
#18	+	+	−	+	−	+
#26	+	+	+	+	−	+
#27	−	+	−	+	+	+
#30	−	+	+	+	−	+
#36-2	−	−	−	+	−	−
#43-1	+	+	−	+	−	+
#43-2	−	+	−	+	−	+
**(b)**						
#16	+	+	+	+	−	+
#16-1	−	+	+	+	+	+
#16-2	+	+	−	+	−	+
#16-3	−	+	−	+	−	+
#18	+	+	−	+	−	+
#43-1	−	+	−	+	−	+
#43-2	+	+	−	+	+	+

(a) Mutation type observed in all T_0_ diploid plants obtained and (b) in all T_1_ segregated lines.

For each target, the presence or absence of sub mentioned type of mutation is indicated with a ‘+’ for presence or ‘−’ for absence, respectively.

### Characterization of the triple mutations induced in the T_1_ lines

**Figure 3:**
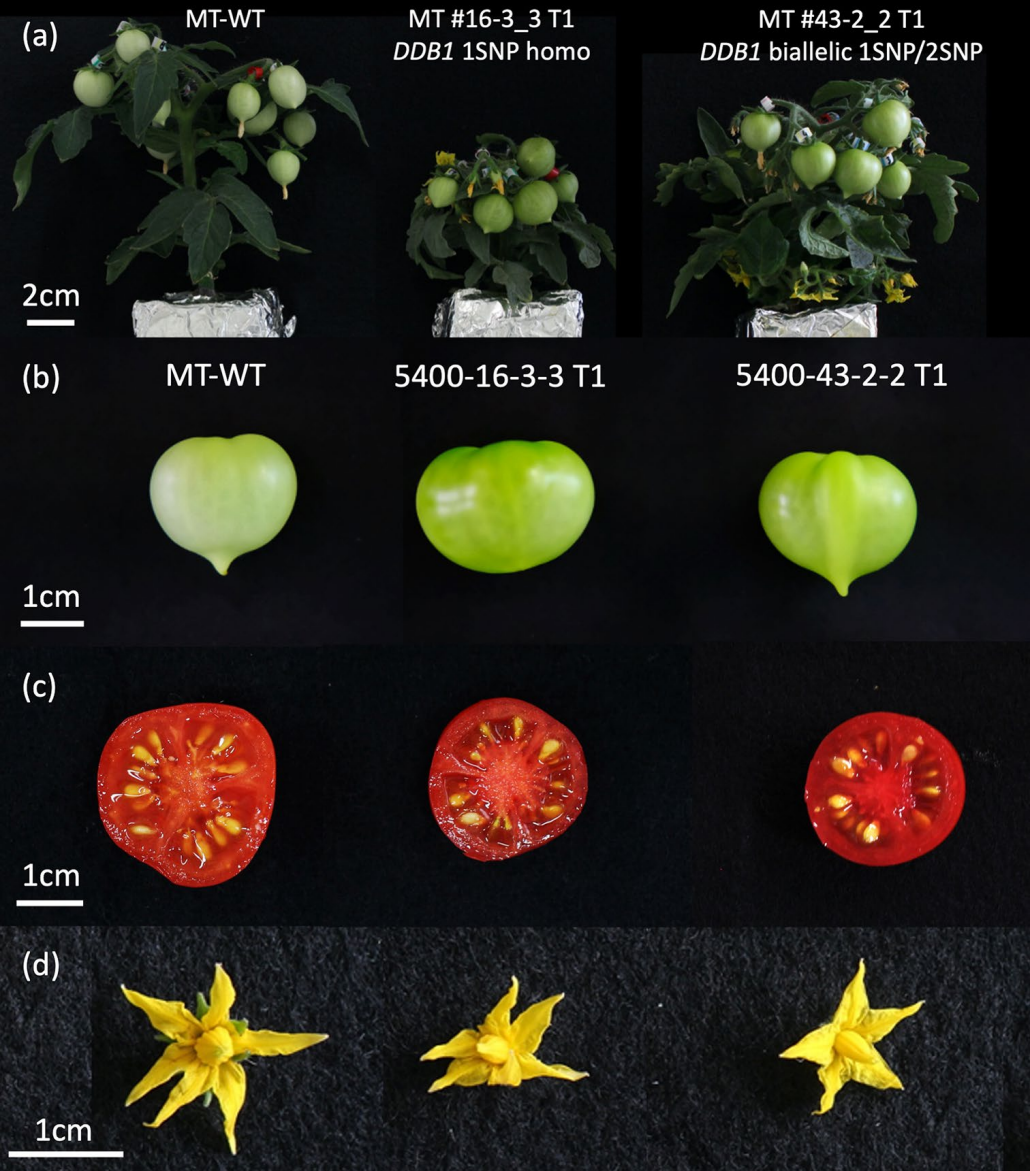
Plant phenotype of segregant generation. (**a**) Six-week-old plants in the T_1_ generation. (**b**) Fruit size and color at the green mature stage (28 days post-anthesis). (**c**) Fruit color at the red ripe stage (12 days post-breaker stage). (**d**) Flower shape and color at the anthesis stage. We noted a similar degree of darkness between the red fruits of the WT and both mutant lines.

**Figure 4:**
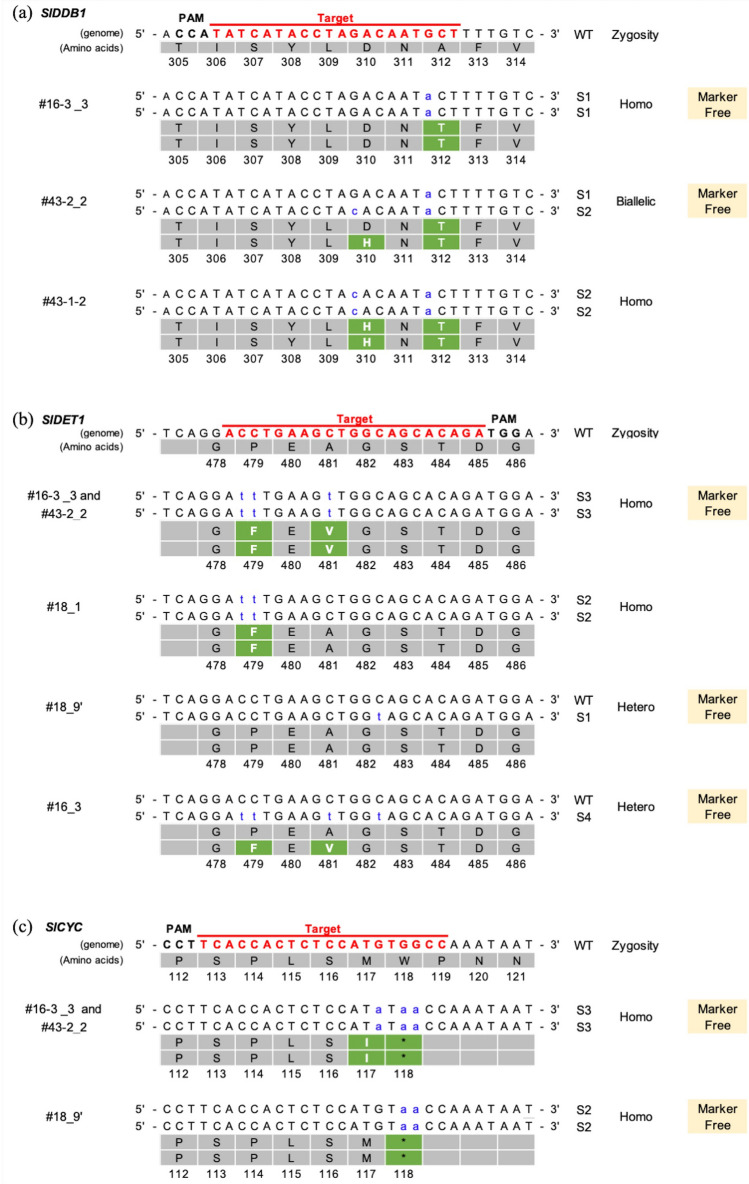
Target AID vectors induce nucleotide and amino acid substitutions in gene-edited tomato plants. Representative substitution spectra and changes in amino acid residues in T_1_ plants. All figures show target sequences, PAM sequences and DNA modifications are in bold red, bold black, and bold blue font, respectively. Numbers under amino acid sequence represent the residue number position in the protein sequence. (**a**) Changes in amino acid residues in DDB1-targeted T_1_ plants; (**b**) changes in amino acid residues in DET1-targeted T_1_ plants. (**c**) Changes in amino acid residues in CYC-B-targeted T_1_ plants. ‘S’ followed by a number correspond to substitution, with the number of nucleotides substituted. The substitutions in amino acid residues were due to the corresponding DNA substitutions in T_1_ plants. The marker gene-free plants are highlighted.

Seven lines descendances providing fruits and seeds are investigated. The seeds of the 7 T_1_ lines (#16, #16-1, #16-2, #16-3, #18, #43-1, #43-2) were grown and observed. A minimum of 6 plant per line were observed, and line #16-1 showed few viable seeds. As expected, all lines show substitutions on all targets, resulting from segregation (Table 2b). In line #18, as the T_0_ generation showed a very high percentage of indels, above 90%, more T_1_ lines were analyzed. Interestingly, none of the lines showed a homozygous indel in *SlDDB1*, suggesting a potential lethal effect of the knockout of this gene. This was also confirmed in heterozygous T_2_ plants from line #18, in which no plants carried a homozygous indel. Only plants showing homozygous or biallelic patterns were used for analysis, which were limited to 2 lines obtained from independent calli. The obtained plants showed the same phenotype as the *hp* mutant as expected, with purple roots, a dwarf morphology and darker green fruit compared to the WT (Fig. 3a,b, Table 3c, Supplementary Fig. S1a). To analyze the impact on photomorphogenesis, T_2_ generation was used. We could observe an impact on photomorphogenesis with smaller hypocotyl on T_2_ mutant lines than WT line, under light and dark conditions (Supplementary Fig. S1b,c), as reported previously on double mutant grown under darkness^18^. The fruits did not show a significantly darker red epidermal color but tended to show a darker red color of the placenta, especially in line #43-2, more noticeable under light (Fig. 3c). As somatic mutation induced by the presence of the construct remained possible, only null segregant plants that did not show the presence of transgene markers were selected for further experiments. The patterns of mutation observed in all T_1_ lines analyzed are summarized in Supplementary Table S3. For *SlDDB1*, Sanger sequencing showed a common pattern of substitution in a homozygous state, where guanine was replaced by adenine at nucleotide 8 383 in lines #16, #16-2, #16-3, #18 and #43-1, resulting in the substitution of Ala by Thr, while in lines #16 and #16-2, a guanine was replaced by a cytidine or thymine at nucleotide 8 377, resulting in the substitution of Asp by His or Tyr, respectively. Interestingly, several plants showed indel in T_1_ which was not noticed in parental plants. Line #43-2 showed one plant with an indel in *SlDDB1 and SlCYC-B*, not visible in the parental plant by NGS analysis, and a biallelic state, carrying both substitutions described above in several other plants, not noticed in the parental plant (Table 2b, Fig. 4a). For *SlDET1*, lines #16 and #16-1 present an indel which was not noticed by NGS sequencing too (Table 2b). It will probably result from a remaining activity of the nCAS9 in plants carrying such substitutions.

**Table 3:** Carotenoid content in µg g^−1^FW of T_1_ and T_2_ segregant null at red ripe fruits and chlorophyll contents of T_2_ segregants at green mature fruits.

Plant lines	Phytoene	Phytofluene	Lycopene	β-Carotene	Lutein	Neoxanthin	Relative total carotenoid
**(a)**							
WT	14.29 ± 3.45	3.91 ± 0.97	30.75 ± 9.50	2.02 ± 0.65	0.38 ± 0.16	0.06 ± 0.03	68.24 ± 17.39
#16-3_3	10.72 ± 0.83*	6.00 ± 0.51*	40.51 ± 7.40	4.19 ± 0.96*	1.06 ± 0.21*	0.20 ± 0.04*	100.03 ± 13.58*
#43-2_2	11.61 ± 0,88*	6.52 ± 0.43*	44.63 ± 3.28*	4.03 ± 0.74*	1.02 ± 0.18*	0.19 ± 0.06*	107.85 ± 4.08*
**(b)**							
WT	15.11 ± 3.33	3.60 ± 0.73	31.28 ± 9.96	1.80 ± 0.47	0.31 ± 0.06	0.05 ± 0.02	66.15 ± 17.75
#16-3_3	9.52 ± 1.26*	2.48 ± 0.36*	38.12 ± 11.50	4.86 ± 1.42*	1.17 ± 0.26*	0.25 ± 0.08*	83.64 ± 24.26
#43-2_2	17.86 ± 4.23	2.73 ± 0.92*	43.39 ± 13.78*	2.34 ± 0.38*	0.65 ± 0.08*	0.08 ± 0.01*	90.37 ± 23.47*

Plant lines	Chlorophyll ‘a’	Chlorophyll ‘b’	Total chlorophyll				
**(c)**							
WT	18.94 ± 2.08	7.46 ± 1.03	26.40 ± 3.04				
#16-3_3	50.81 ± 4.25*	18.53 ± 1.31*	69.34 ± 5.52*				
#43-2_2	37.67 ± 3.38*	14.14 ± 1.63*	51.81 ± 5.01*				

(a) and (b) The carotenoid content was measured in the fruit pericarp by HPLC at the breaker-stage + 12 days (12 DPB) in T_1_ null plants and T_2_ segregants, respectively. Concentrations are expressed in µg g^−1^FW. (c) The chlorophyll content was measured in the fruit pericarp by spectrophotometry at the green mature stage (28 DPA) in T_2_ segregants. Concentrations are expressed in µg g^−1^FW. Statistical analysis with T-test independent variable was performed between the mutated lines to the control line for T_1_ and T_2_ for carotenoid content and for chlorophyll content, with significant differences were considered to exist at p < 0.05.

*Means significantly different at 0.05.

In the case of *SlDET1*, 3 patterns of substitutions in a homozygous state could be observed, all involving the replacement of cytidine with thymine. The double substitution of nucleotides 19,850 and 19,851 resulted in the substitution of Pro by Phe in line 18; a triple substitution at nucleotide 19,857 resulting in the substitution of Asp by Val as the 3rd substitution in lines #16, #16-3, #43-1 and #43-2; and a quadruple substitution resulting in a silent mutation in a codon for Gly at nucleotide 19,861 for line #16-1. Other patterns of mutations were found, but because they were present in a heterozygous form in a single plant, they were excluded from the analysis (Fig. 4b).

Finally, for *SlCYC-B*, only 2 patterns involving the substitution of guanine by adenine could be observed in a homozygous state. A double substitution led to the formation of a stop codon at nucleotides 353 and 354 and a triple substitution at nucleotides 351, 353 and 354 resulted in the substitution of Met by Ile and the formation of a stop codon immediately thereafter (Fig. 4c). The interesting phenotype reported for mutations in *SlCYC-B*^20^ of darker petals could not be observed (Fig. 3d).

### Evaluation of carotenoid accumulation in the T_1_ lines

After selection, only 2 independent callus lines were selected for carotenoid accumulation analysis in the T_1_ generation on the basis of being triple-substituted mutant lines. Five fruits per plant was harvested. In each independent T_1_ line selected, only 1 plant showed null segregation and was used for segregant analysis to avoid the somatic mutation effect due to the presence of the transgene in T_1_ plants (Supplementary Table S4). Other lines, such as line #43-2_7′, were not used due to a premature death before obtaining seeds. Fruits were harvested at 12 days post-breaker stage (DPB), and only the pericarp was retained to prevent bias resulting from the whiter columella color in the WT. The total carotenoid content was measured by HPLC and adjusted to the lycopene standard for calculation. As each of the null segregant lines #16-3_3 T_1_ and #43-2_2 T_1_ presented a different mutation in *DDB1*, with a biallelic version of the gene occurring in #43-2_2 (Fig. 4a), differentiation of the carotenoid composition and accumulation was expected. Line #43-2_2 showed a 2nd SNP at nucleotide 928 only in the heterozygous state, and as plants with no mutation at this position showed a similar phenotype to the heterozygotes (Fig. 3c), this mutation was considered recessive and did not affect the results under biallelic conditions. The results of the analysis in the T_1_ lines showed an almost two-fold increase in lycopene in lines #43-2_2, whereas #16-3_3 showed an increase of approximately 30% (Table 3a). The accumulation of β-carotene was expected to be reduced compared to that in the WT, resulting in an expected disruption of the CYC-B enzyme. Interestingly, we observed an increase in β-carotene in #16-3_3 of more than two-fold in the T_1_ generation. In the #43-2_2 line, the β-carotene content remained stable compared to that in the WT. Same tendency of accumulation could be observed in T_2_ generation, with a total carotenoid, lycopene and β-carotene higher accumulation in mutated lines compare to the WT (Table 3b). However, at the green stage, fruits from line #16-3_3 showed a darker color (Fig. 3c), resulting from a higher content of chlorophyll, suggesting a greater increase in carotenoids compared with line #43-2_2 (Table 3c). Regarding the upstream carotenoid pathway, both mutated lines showed greater accumulation of phytofluene in the T_1_ line. In the downstream portion of the lycopene pathway, only line #16-3_3 showed an increase in the accumulation of lutein and neoxanthin.

## Discussion

Our results confirm that Target-AID is an efficient tool for inducing multiple-gene cytidine substitutions in tomato, with a substitution ratio higher than 40% of total number of reads without indel in all three targets simultaneously for all lines analyzed in the T_0_ generation, and no off-targets were detected (visualized in Fig. 2, Supplementary Table S1b). As reported previously, Target-AID tends to produce C to T and G to A substitutions^3,12^. As tomato is routinely subjected to transformation with standard protocols developed over decades^25,26^, the ratio of regeneration and transformation is known to follow standard patterns close to 10%. Among the 12 regenerated plants, 11 presented substitutions at all three targets simultaneously, representing about 91.6% of efficiency in regeneration. This ratio was much higher than that obtained in previous studies for multiplex targeting by non-modified Cas9 system. A study on the carotenoid pathway in tomato, showed more variable results depending on the target in the pathway (0 to 95.83% per target, with only 8.33% carrying more than 2 target mutations)^27^. Same observation was also reported on parthenocarpy trait, using non modified Cas9 system (11 to 65%)^28^. However, the promoter of the Cas protein or gRNA influences the efficiency of edition^28^, as observed for codon-optimized versions such as CDA *Arabidopsis thaliana*-optimized versions^12^.

The nCas9 protein generates single-strand breaks, and the UGI protein decreases indel formation after replication^3^, resulting in an indel rate lower than that coming from the use of the conventional Cas9 system. The Cas9 protein is mainly used to generate indels after the fixation of a lesion, and the most high-efficiency edition results in frameshift mutations^27,29,30^. In the case of Target-AID, the segregation of the generated mutations tends to produce lines carrying substitutions instead of indels in either plant or animal cells^9,12^. In our study, the ratio of indels varied from more than 90% of total reads resulting from NGS sequencing for *SlDDB1* in line #18 to 0% for *SlDET1* in all lines, with an average of 15% to 25% (Supplementary Table S1). This was observed upon direct PCR product sequencing in the T_0_ generation, with very low histogram peaks mix noticed downstream of the indel site on Sanger reads (Supplementary Fig. S2), coming from the presence of few amplificons of the DNA strand carrying indel, resulting in a potential error of interpretation as a noise background. This low amount of indel can also be noticed by the few amounts of heterozygote plants carrying an indel in the next generation (Supplementary Table S3).

The mutations in *SlDDB1* and *SlDET1* originally described in previous publications regarding *hp* tomato mutants^17–19^, resulted in SNPs that do not induce total knockout of the gene, probably due to lethal effects in the homozygous condition. As indels were present only in the heterozygous condition for *SlDDB1* and *SlDET1* (Supplementary Table S3), this could have resulted from a potential deleterious effect of the knockout of these genes*.* In the case of *SlDET1*, the original *Slhp2* mutation led to alternative splicing, inducing a deletion of three amino acids in exon 11, resulting in a truncated NLS^19^. However, this mutation was described as an incomplete knock-out mutation, with approximately 10% of the mRNA being correctly spliced. No indel allele mRNA of *SlDDB1* has been reported previously. In our case, the absence of homozygous plants for an indel in *SlDDB1* or *SlDET1* could be explained by the necessity for a small amount of functional protein, as no T_2_ homozygous indel lines could be obtained from line #18, which showed the highest segregation of indels.

Two alleles of *SlDDB1* could be obtained, with greater availability of the single SNP version. For *SlDDB1*, one mutation was located 4 bp from the original *Slhp1* mutation, resulting in the substitution of the subsequent amino acid compared to the original mutation^17^. The substitution of alanine by threonine was located at a position that is not strongly conserved compared to the previous asparagine to tyrosine substitution in *Slhp1* (Supplementary Fig. S3)*.* As each amino acid belong to a different group, exhibiting a hydrophobic side chain or an uncharged polar chain, respectively, these substitutions might induce a change of conformation of the protein. However, the substitution of aspartic acid by histidine at amino acid 315 in line #42-2-2 occurred in a strongly conserved domain, potentially resulting in a strong effect on plant growth, explaining the difficulty of obtaining a homozygote for this position (Supplementary Fig. S3). A potentially stronger effect on carotenoid accumulation compared to line #16-3-3, with a codominant effect, may be considered. The impact of this mutation should be observed in segregating homozygotes in the T_2_ population.

Impact on photomorphogenesis could be observed with smaller hypocotyl of double mutant grown under light and dar conditions (Supplementary Fig. S1b,c) as reported previously in tomato^18^. But analysis of impact of each mutation on segregation generation should be performed to confirm if we have an additional effect of both mutations on the plant growth, as reported^18^.

The generation of a truncated protein is possible by the introduction of a stop codon, as in our example involving *SlCYC-B*. The original *Slog*^*c*^ mutant has been reported to exhibit an insertion resulting in a frameshift, showing the nonlethal effect of knocking out the gene. As the *SlCYC-B* target resulted in a stop codon and, thus, a predicted truncated protein, the mutant should show a darker orange flower phenotype compared to the WT, as described in *Slog* mutants^20^. Interestingly, we could not clearly observe such a phenotype of tawny orange flowers (Fig. 3). The *Sl*CYC-B protein also functions as a neoxanthin synthetase in addition to a chromoplastic lycopene-β-cyclase, which results in the transformation of violaxanthin to neoxanthin^31^. In our loss of function of *SlCYC-B* lines, we do not observe a decrease in β-carotene or neoxanthin contents as expected. Interestingly, we can observe an increase in both lutein content, β-carotene and neoxanthin (Table 3a,b). Such observation of no decrease of β-carotene in *Sl*CYC-B defective lines has been already reported in the Micro-Tom background^32^. This can probably be explained by the Micro-Tom genotype, as 2 different populations, MT Brazil^32^ and MT Japan, in our case, present a mutation in *SlCYC-B* without a phenotype of modification in lycopene accumulation.

In conclusion, Target-AID is an efficient tool for inducing multiple-gene substitutions in tomato, which are identified in a mutant population. The conventional Cas9 technique can cause a potential problem regarding the lethal effect of gene knock-out caused by indel mutations, as observed for *SlDDB1* and *SlDET1.* Although the generation of a single target mutation has already been demonstrated in the *Sletr1* and *Sldella*^12^ genes, this is the first report of the introduction of substitutions among multiple genes in tomato. This technique will be a useful tool for the engineering of breeding traits controlled by multiple genes in elite lines of tomato. It can also be used to create new alleles from spontaneous mutations discovered in other mutant populations, including those obtained through spontaneous, gamma- or X-ray or Ethyl methanesulfonate (EMS) mutagenesis, such as the original *Sletr1*^33^ and *Sldella*^34^ mutants. New technologies for targeted replacement in the genomic DNA space and the introduction of new codons are still under development using CRISPR-Cas technology such as Prime Editing^35^. However, as such technology still shows a very low efficiency, the Target-AID is currently one alternative whereby breeders can introduce allelic variations in multiple targets in a single line and single generation.

## Materials and methods

### Vector construction

The vector construct used in this study was derived from a previous study^12^ but was modified by the insertion of the UGI fragment and amplified by PCR from pScI_dCas9-CDA-UL (Addgene #108551)^14^ using the following primers: kn2052, CTAAGAAGAAACGTAAAGTAgggcCcATGACCAACCTTTCCGAC and kn2053, agctgggaggcctggatcAgGgctatgcaaccagtccTAGCATC. The fragment was then inserted into ApaI-digested pDicAID_nCas9-PmCDA_NptII by Gibson assembly to obtain pDicAID_nCas9-CDA-UGI-LVA_NptII.

The target guide cassette was replaced as described below. Each of the target sgRNA sequences for *SlDDB1*, *SlDET1* and *SlCYC-B* was individually inserted into pDicAID between the *At*U6 promoter and the chimeric sgRNA scaffold (presenting an AscI site in the 5′-terminal region and an MluI site in the 3′-terminal region of the gRNA) by a PCR method and then circularized by Gibson assembly. Each resulting target sgRNA expression unit consisted of the *At*U6 promoter with an I-SceI-AscI site in the 5′-terminal region and a target-specific sgRNA with an MluI-SpeI-I-SceI site in the 3′-terminal region. New multiplexing plasmids were generated by double digestion as follows: MluI and SpeI double digestion for target 1(*SlDDB1*) to open the vector and AscI and SpeI double digestion for target 2(*SlDET1*) to release the fragment for extraction by gel purification. The products were ligated by recombination at the AscI-digested site with MluI and then transferred into highly competent *E. coli*. The same process was repeated for target 3(*SlCYC-B*) using the previous plasmid construct obtained from the ligation of the target 1 and target 2 products. Primer list used for the construction of the plasmid can be found in supplementary data (Supplementary Table S5).

### Plant transformation and culture

The tomato (*Solanum lycopersicum*) cv. Micro-Tom Japan was used in this study, which was provided by the NBRP tomato program at the University of Tsukuba. The vector construct was introduced by *Agrobacterium* transformation with slight modification^25,26^. Transgenic diploid plants were selected by kanamycin resistance. The plants and their descendants (T_1_ and T_2_ lines) were grown in a culture chamber under an LED photosynthetic photon flux of 270 µmol m^−2^ s^−1^ light (16 h/8 h) at a constant temperature of 25 °C. About 151 explants were infected and only 13 diploid plants were successfully regenerated and analyzed in further experimentations.

Photomorphogenesis experimentation has been conducted on T_2_ generation under same light condition as growing plants, or under total darkness, for 7 days. Seeds were treated with a bleach for 15 min followed by 4 washes with autoclaved tap water and transferred on ½ MS medium in Magenta box. Box were transferred for 2 days under darkness, then for 5 days under light for light experiment, or 6 consecutive days in dark followed by one day in light condition. Hypocotyl length were measured using digital caliper (Mitutoyo, Japan).

### PCR and sequencing analysis

For PCR amplification, the primers used in this study are listed in Supplementary Table S5. For NGS sequencing, each target locus was amplified using PrimeSTAR GXL (Takara, Japan) following the manufacturer’s protocol, and the second-round PCR products were extracted from 0.8% agarose gels under blue LED light to prevent PCR product degradation. For Sanger sequencing, targets were amplified using KOD FX Neo (Takara, Japan) according to the manufacturer’s protocol, and the PCR product was directly purified using the FastGene Gel/PCR Extraction kit (Nippon Genetic, Japan).

### NGS analysis

The design of the target was conducted as reported in our previous study on the Target-AID system^12^ using CCTop (https://crispr.cos.uni-heidelberg.de/index.html), and selection was performed according to the proximity of the original mutation described for *SlDDB1*^17,18^ and *SlDET1*^19^ and the potential for the generation of a stop codon in *SlCYC-B*^20^. Off-target associations were selected based on their higher scores. Both target and off-target effects were analyzed in 7 independent T_0_ lines, which were then used for the production of T_1_ seeds. The fragment containing the target region (1 kb) was firstly isolated by PCR reaction from genomic DNA, using the first primer set. After gel verification, to obtain an adaptor-containing amplicon (~ 0.5 kb), a second round of nested PCR was conducted using the first product as a template and a second pair of primers containing adaptor sequences. Expected products were extracted after separation in 0.8% agarose gels and band extraction using a FastGene Gel/PCR Extraction kit (Nippon Genetic, Japan). The concentration and quality of the samples were determined using the KAPA Library Quantification kit for Illumina (Kapa Biosystems, Roche, CH) on a BioRad CFX96 qPCR system (BioRad, CA, USA). Approximately 50 ng of the product per sample was bulked for analysis. The primers used in this experiment are listed in Supplementary Table S5. The amplicons were labelled using NEBNext Multiplex Oligos for Illumina (Index Primer Set 1 and Dual Index Primer Set 1) (New England BioLabs, MA, USA). Deep sequencing was performed on a MiSeq sequencing system (Illumina, CA, USA), and more than 200 000 paired 300 bp reads per sample were obtained on average. Data analysis was performed using Geneious Prime 2019 (Biomatters, Auckland, New Zealand (https://www.geneious.com)). The sequence reads were paired and trimmed using the BBDuk Adapter/quality Trimming Version 38.37 plug-in from Brian Bushnell, based on the following settings: Trim Adapter with all libraries, Kmer length: 37; maximum substitutions: 1; maximum substitutions + INDELs: 0; trim low quality: both ends; minimum quality: 30; trim adapters based on paired read overhangs: minimum overlap: 20; discard short reads, minimum length: 200 bp. Paired and trimmed samples were mapped to the reference using Geneious mapper with the following settings: sensitivity: highest sensitivity; find structural variants, short insertions, and deletion of any size; minimum mapping quality: 30; trim paired read overhangs; only map paired reads that map nearby; minimum support for structural variant discovery: 2 read, include insertions in structural variants. Variant calling was performed with the following settings: minimum coverage: 10; minimum variant frequency: 0.05; maximum variant p-value: 10-3: 3 (0.1% to see variant by chance); p-value calculation method: approximate; advanced: merge adjacent variations. Both in- and off-target mutation frequencies were calculated using the numbers of reads with DNA modifications per total reads sequenced, directly obtained by variant calling. The logo Fig. 1 was produced using the Logomaker plug-in, under Python software^36^ (https://github.com/jbkinney/logomaker).

### Analysis of the mutation spectrum in T_0_ and descendant transgenic plants by Sanger sequencing

To analyze the mutation spectrum of DNA modifications, in combination with NGS sequencing, target regions were amplified by PCR using specific primers (Supplementary Table S5) and genomic DNA extracted from the leaves of WT and transgenic plants (T_0_, T_1_) as templates. For the T_0_ lines, in the presence of chimeric plants at a high ratio, the amplified PCR fragments were cloned into the pGEM-T easy plasmid (Promega, USA) after Taq treatment at 72 °C for 30 min, which was then cloned into the DH5*α* strain (Toyobo, Japan) for sequencing on the ABI 3130xl system (Applied Biosystems, CA, USA). The bacteria were plated on LB agar plates containing 50 µg ml^−1^ ampicillin. Approximately 10 colonies for each target were picked using blue/white selection and confirmed by the PCR amplification of the appropriate fragment from each colony. Their insert sequences were determined by the Sanger method using target-specific primers. The T_1_ and T_2_ generation were sequenced directly after purification using the FastGene Gel/PCR Extraction kit (Nippon Genetic, Japan).

The conservation scores of the amino acid sequences of *SlDDB1* and *SlDET1* were determined on the Consurf website server^37^, using the PBD ID of the protein and maintaining the standard settings.

### Carotenoid and chlorophyll analysis

Carotenoid analysis was performed as follows. Five red fruits per plant were harvested at 12 DPB, and the pericarp was frozen in liquid nitrogen and ground to a fine powder using a Multi Beads Shocker (Yasui Kikai, Japan). The powder was stored at − 80 °C for multiple purposes. For T_1_ experimentation, only 1 plant of edited line could be analyzed as null segregant, divided in 3 individuals technical (n = 3) replicates and 5 plants for WT line (n = 15). For T_2_ generation, 4 plants for WT line, line #16-3_3 (n = 12) and 3 plants for line #43-2_2 (n = 9) have been used, divided in 3 technical replicates for each plant. To perform carotenoid extraction, 100 mg of powder was used, and extraction was conducted following the petal carotenoid protocol^38^ with some modifications. Before extraction, the samples were placed in a 2 ml tube with 2 zirconium beads and shaken for 30 s to break up any large powder agglomerates. Then, 30 μl of 1 M Tris–HCl (pH 7.6–8) and 200 μl of acetone was added, followed by mixing by hand. Then, 500 μl of diethyl ether was added, followed by mixing by hand and centrifugation for 1 min at 10,000 rpm using a table centrifuge. The upper organic phase was then transferred to a new tube, and the previous steps were repeated until the total discoloration of the organic phase. Thereafter, 500 μl of 5 mM Tris–HCl (pH 7.6–8) and a few drops of saturated saline solution were added to the collected organic phase, followed by mixing by hand and brief centrifugation. The lower aqueous phase was removed by pipetting and discarded, and the previous step was repeated 3 times to remove the remaining acetone and water present in the organic phase. The upper phase was then transferred to a new tube, and half of the volume of 10% KOH–MeOH (W/V) is added, followed by mixing by hand. The mixture was kept for 1 to 2 h in the dark to saponify the ester groups. Then, 1 ml of distilled water was added to the tube, followed by mixing and centrifugation. The lower aqueous phase was then removed, and the previous step was repeated 3 times. The upper phase was transferred to a new tube, and 300 μl of diethyl ether was added to recover as much of the carotenoid extract as possible. In the new tube, 100% EtOH was added to the total organic volume at a rate of 20 to 30%, followed by mixing. The samples were dried under a vacuum desiccator for 1 h at RT, and the pellet was checked to ensure that it was correctly dried. The samples were stored at − 80 °C until analysis. For analysis, each extract was analyzed by means of high-performance liquid chromatography (HPLC) (X-LC, JASCO, Tokyo, Japan)^39^ with a photodiode array detector (X-LC 3110MD, JASCO) under the following conditions: column, YMC Carotenoid (100 mm × 4.6 mm i.d., 3 μm; YMC, Kyoto, Japan); solvent A, methanol (MeOH)/methyl tert-butyl ether (MTBE)/H2O = 95:1:4 (v/v/v); solvent B, MeOH/MTBE/H2O = 25:71:4; gradient, 0/100, 5/100, 38/0 (min/% A); flow rate, 1.0 ml/min; column temperature, 35 °C; UV–Vis monitoring range, 200–600 nm. The contents were calculated according to the total peak area of the HPLC chromatograms at a wavelength of 450 nm for lycopene, β-carotene, lutein and neoxanthin. Phytoene and phytofluene were measured at 285 nm and 345 nm, respectively. Total carotenoids were expressed as the lycopene equivalents and neoxanthin as lutein equivalent. Lycopene, β-carotene, lutein, phytoene and phytofluene were measured with the corresponding standards. All other carotenoid compounds are expressed as lutein equivalents of the tissue.

The chlorophyll content was analyzed as described by Nagata and Yamashita^40^. Five green fruits per plant were harvested at 28 DPA, from 3 plants per line, and the pericarp was frozen in liquid nitrogen and ground to a fine powder using a Multi Beads Shocker (Yasui Kikai, Japan). The powder was stored at − 80 °C for multiple purposes. For chlorophyll extraction, 200 mg of powder was used per biological sample, with 3 technical replicates (n = 9), and extraction was performed using 2 ml of acetone-hexane (4:6 v/v) in a 5 ml tube, which was vortexed and then sonicated for 20 s. All samples were kept on ice and protected from light to prevent degradation. The samples were briefly centrifuged, 700 μl of the organic phase was transferred to a quartz cuvette, from which the absorbance at 645 (A_645_), 663 (A_663_) and 750 (A_750_) nm was read on a spectrophotometer (SpectraMax M2, Molecular Devices, USA). The contents of chlorophyll ‘a’ and chlorophyll ‘b’ were calculated using the following equations in μg g^−1^FW.:

$${\text{Chlorophyll }}{\text{`a'}} = \left( {0.999{\text{A}}_{663} - 0.0989{\text{A}}_{645} } \right) \times 100$$

$${\text{Chlorophyll }}{\text{`b'}} = \left( { - \;0.328{\text{A}}_{663} + 1.77{\text{A}}_{645} } \right) \times 100$$

### Statistical analysis

Statistical analysis was performed with IBM SPSS Statistics 25 (SPSS Inc., Chicago, USA) via independent-samples T test and a significant difference was considered to occur at p < 0.05.

Hypocotyl size figures (Supplementary Fig. S1b,c) were produced on R software, using R package *ggplot 2* version 3.3.2^41^ (https://ggplot2.tidyverse.org).

## Supplementary information

Supplementary Information.

## Data Availability

Short read sequence data have been deposited at the NCBI Sequence Read Archive (SRA) under the accession codes PRJNA633920. The remaining data that support the findings of this study are available from the corresponding author upon reasonable request.

## References

[1] Hsu PD, Lander ES, Zhang F (2014). Development and applications of CRISPR-Cas9 for genome engineering. Cell.

[2] Komor AC, Kim YB, Packer MS, Zuris JA, Liu DR (2016). Programmable editing of a target base in genomic DNA without double-stranded DNA cleavage. Nature.

[3] Nishida K (2016). Targeted nucleotide editing using hybrid prokaryotic and vertebrate adaptive immune systems. Science.

[4] Nishimasu H (2018). Engineered CRISPR-Cas9 nuclease with expanded targeting space. Science.

[5] Zong Y (2017). Precise base editing in rice, wheat and maize with a Cas9-cytidine deaminase fusion. Nat. Biotechnol..

[6] Sakata RC (2019). A single CRISPR base editor to induce simultaneous C-to-T and A-to-G mutations. bioRxiv.

[7] Lu X (2018). Optimized Target-AID system efficiently induces single base changes in zebrafish. J. Genet. Genomics.

[8] Ma Y (2018). Highly efficient and precise base editing by engineered dCas9-guide tRNA adenosine deaminase in rats. Cell Discov..

[9] Sasaguri H (2018). Introduction of pathogenic mutations into the mouse *Psen1* gene by Base Editor and Target-AID. Nat. Commun..

[10] Li C (2018). Expanded base editing in rice and wheat using a Cas9-adenosine deaminase fusion. Genome Biol..

[11] Negishi K (2019). An adenine base editor with expanded targeting scope using SpCas9-NGv1 in rice. Plant Biotechnol. J..

[12] Shimatani Z (2017). Targeted base editing in rice and tomato using a CRISPR-Cas9 cytidine deaminase fusion. Nat. Biotechnol..

[13] Rees HA, Liu DR (2018). Base editing: precision chemistry on the genome and transcriptome of living cells. Nat. Rev. Genet..

[14] Banno S, Nishida K, Arazoe T, Mitsunobu H, Kondo A (2018). Deaminase-mediated multiplex genome editing in *Escherichia coli*. Nat. Microbiol..

[15] Carvalho RF (2011). Convergence of developmental mutants into a single tomato model system: ‘Micro-Tom’ as an effective toolkit for plant development research. Plant Methods.

[16] Willcox JK, Catignani GL, Lazarus S (2003). Tomatoes and cardiovascular health. Crit. Rev. Food Sci. Nutr..

[17] Lieberman M, Segev O, Gilboa N, Lalazar A, Levin I (2004). The tomato homolog of the gene encoding UV-damaged DNA binding protein 1 (*DDB1*) underlined as the gene that causes the *high pigment-1* mutant phenotype. Theor. Appl. Genet..

[18] Liu Y (2004). Manipulation of light signal transduction as a means of modifying fruit nutritional quality in tomato. Proc. Natl. Acad. Sci. U.S.A..

[19] Mustilli AC, Fenzi F, Ciliento R, Alfano F, Bowler C (1999). Phenotype of the tomato *high pigment-2* mutant is caused by a mutation in the tomato homolog of *DEETIOLATED1*. Plant Cell.

[20] Ronen G, Carmel-Goren L, Zamir D, Hirschberg J (2000). An alternative pathway to β-carotene formation in plant chromoplasts discovered by map-based cloning of *Beta* and *old-gold* color mutations in tomato. Proc. Natl. Acad. Sci..

[21] Hwang I, Kim Y, Han J, Nou IS (2016). Orange color is associated with *CYC-B* expression in tomato fleshy fruit. Mol. Breed..

[22] Mohan V, Pandey A, Sreelakshmi Y, Sharma R (2016). Neofunctionalization of chromoplast specific lycopene beta cyclase gene (*CYC-B*) in tomato clade. PLoS ONE.

[23] Isaacson T, Ronen G, Zamir D, Hirschberg J (2002). Cloning of *tangerine* from tomato reveals a carotenoid isomerase essential for the production of β-carotene and xanthophylls in plants. Plant Cell.

[24] Peters JL, Schreuder MEL, Verduin SJW, Kendrick RE (1992). Physiological characterization of a high-pigment mutant of tomato. TGC Rep..

[25] Gupta S, Van Eck J (2016). Modification of plant regeneration medium decreases the time for recovery of *Solanum lycopersicum* cultivar M82 stable transgenic lines. Plant Cell. Tissue Organ Cult..

[26] Shikata M, Ezura H (2016). Micro-Tom tomato as an alternative plant model system: mutant system: mutant collection and efficient transformation. Plant Signal Transduct. Methods Protoc..

[27] Li X (2018). Lycopene is enriched in tomato fruit by CRISPR/Cas9-mediated multiplex genome editing. Front. Plant Sci..

[28] Hashimoto R, Ueta R, Abe C, Osakabe Y, Osakabe K (2018). Efficient multiplex genome editing induces precise, and self-ligated type mutations in tomato plants. Front. Plant Sci..

[29] Nonaka S, Arai C, Takayama M, Matsukura C, Ezura H (2017). Efficient increase of γ-aminobutyric acid (GABA) content in tomato fruits by targeted mutagenesis. Sci. Rep..

[30] Li R (2018). Multiplexed CRISPR/Cas9-mediated metabolic engineering of γ-aminobutyric acid levels in *Solanum lycopersicum*. Plant Biotechnol. J..

[31] Bouvier F, D’Harlingue A, Backhaus RA, Kumagai MH, Camara B (2000). Identification of neoxanthin synthase as a carotenoid cyclase paralog. Eur. J. Biochem..

[32] Sestari I (2014). Near-isogenic lines enhancing ascorbic acid, anthocyanin and carotenoid content in tomato (*Solanum lycopersicum* L. cv Micro-Tom) as a tool to produce nutrient-rich fruits. Sci. Hortic..

[33] Okabe Y (2011). Tomato TILLING technology: development of a reverse genetics tool for the efficient isolation of mutants from micro-tom mutant libraries. Plant Cell Physiol..

[34] Shinozaki Y (2018). Identification and functional study of a mild allele of *SlDELLA* gene conferring the potential for improved yield in tomato. Sci. Rep..

[35] Anzalone AV (2019). Search-and-replace genome editing without double-strand breaks or donor DNA. Nature.

[36] Tareen A, Kinney JB (2019). Logomaker: beautiful sequence logos in Python. Bioinformatics.

[37] Ashkenazy H, Erez E, Martz E, Pupko T, Ben-Tal N (2010). ConSurf 2010: calculating evolutionary conservation in sequence and structure of proteins and nucleic acids. Nucleic Acids Res..

[38] Kishimoto S, Sumitomo K, Yagi M, Nakayama M, Ohmiya A (2007). Three routes to orange petal color via carotenoid components in 9 compositae species. J. Jpn. Soc. Hortic. Sci..

[39] Liu H, Kishimoto S, Yamamizo C, Fukuta N, Ohmiya A (2013). Carotenoid accumulations and carotenogenic gene expressions in the petals of *Eustoma grandiflorum*. Plant Breed..

[40] Nagata M, Yamashita I (1992). Simple method for simultaneous determination of chlorophyll and carotenoids in tomato fruit. Nippon Shokuhin Kogyo Gakkaishi.

[41] Wickham H (2016). ggplot2: Elegant Graphics for Data Analysis.

