# Supplementary material for: Multiple gene substitution by Target-AID base-editing technology in tomato
Source: Sci Rep. 2020 Nov 24;10:20471. doi: 10.1038/s41598-020-77379-2 (PMC7686336; doi:10.1038/s41598-020-77379-2)
Supplement: Supplementary file 1 — Supplementary Information. [file 41598_2020_77379_MOESM1_ESM.pdf]

Supplementary information

Title:

Multiple gene substitution by Target-AID base-editing technology in tomato

Johan Hunziker[1], Keiji Nishida[2], Akihiko Kondo[2], Sanae Kishimoto[3], Tohru Ariizumi[4] Hiroshi Ezura[4, 5*]

Affiliations:

[1] Graduate School of Life and Environmental Sciences, University of Tsukuba, Tsukuba, Japan. [2] Graduate School of Science, Technology and Innovation, Kobe University, Kobe, Japan. [3] Institute of Vegetable and Floricultural Science, NARO, Tsukuba, Ibaraki, Japan.[4] Faculty of Life and Environmental Sciences, University of Tsukuba, Tsukuba, Japan. [5]Tsukuba Plant Innovation Research Center, University of Tsukuba, Tsukuba, Japan

*Corresponding author

e-mail : ezura.hiroshi.fa@u.tsukuba.ac.jp

tel: (81) 029-853-7263

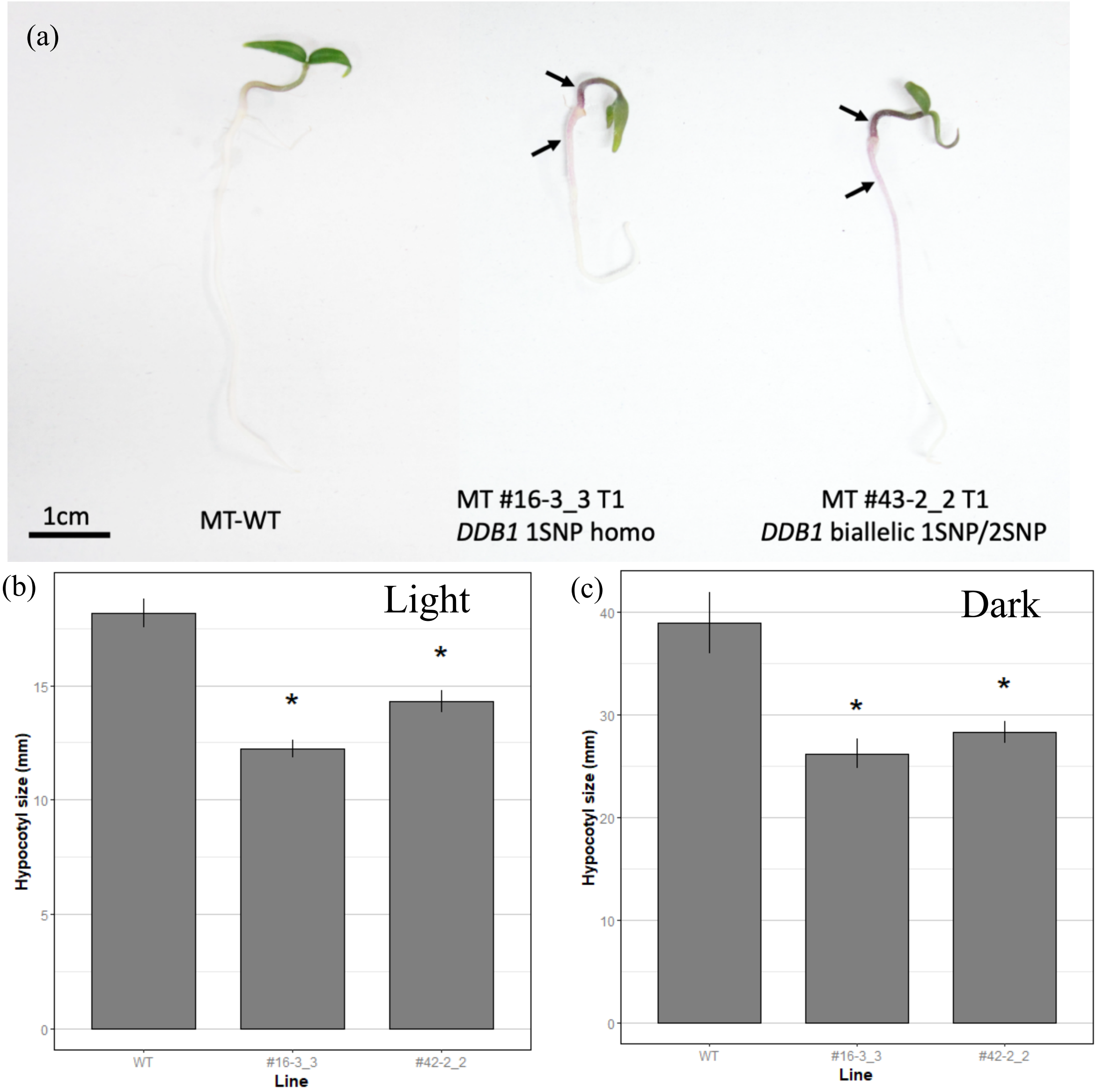

Supplementary Fig. S1

Photomorphogenesis of segregant T_2 generation. (a) We can observe presence of purple hypocotyl and roots in edited lines, from the accumulation of anthocyanin, resulting from the mutation in *SlDDB1* and *SlDET1*. Arrows points the phenotype of anthocyanin accumulation. (b) and (c) Hypocotyl length in millimetre of 7 days old WT and T_2 segregant grown under light and dark condition, respectively. Error bars indicate SD and * indicate significantly different at 0.05 with t-test.

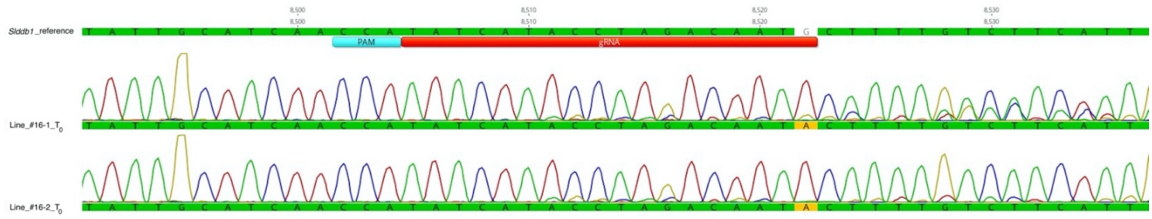

Supplementary Fig. S2 Sanger sequencing histogram of the direct PCR product sequencing of Sl*ddb1* targets in the T_0 generation. We observed a small peak similar to the background noise from the target site, resulting from a frameshift from this position due to an indel.

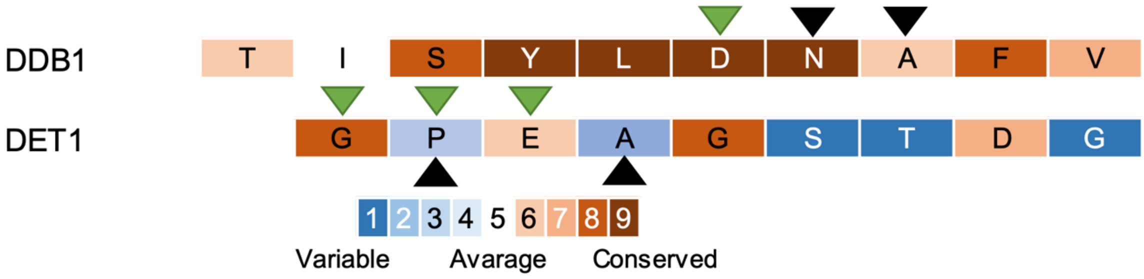

Supplementary Fig. S3 Conservation of target protein regions in SlDDB1 and SlDET1 according to Consurf. The colour scale represents the degree of conservation of the amino acids, with a warmer colour (brown) indicating a more conserved amino acid and a cooler colour (blue) indicating a more variable occurrence of the amino acid. The black triangles represent amino acids modified by Target-AID. A green triangle represents the original *hp1* (*Slddb1*) and *hp2* (*Sldet1*) mutations.

Supplementary Table S1 Deep sequencing analysis of on- and off-target sites induced by Target-AID in tomato.

(a)

SlDDB1 ON/OFF Target

	SNV position		-30	-29	-28	-27	-26	-25	-24	-23	-22	-21	-20	-19	-18	-17	-16	-15	-14	-13	-12	-11	-10	-9	-8	-7	-6	-5	-4	-3	-2	-1	1	2	3	4	5	6	7	8	9	10	11	12
ON Target (*DDB1*)	Sequence		T	G	A	A	G	A	C	A	A	A	A	G	C	A	T	T	G	T	C	T	A	G	G	T	A	T	G	A	T	A	T	G	G	A	A	C	T	A	C	G	T	T
Solyc02g021650: CDS	reads	Indel																																								
WT	416 982	0																																								
Line #16	255 890	DEL 48.4													T 51.5																											
Line #16-1	219 418	INS 26.4													T 40.5						G 5.1																							
		DEL 16.6																																								
Line #16-2	553 616	DEL 15.7													T 83.9																											
		DEL 16.0																																								
Line #16-3	488 320	DEL 12.2													T 66.1						G 5.1																							
		DEL 15.2																																								
Line #18	445 898	INS 93.6													T 68.8						G 48.8																							
		DEL 96.5																																								
Line #43-1	384 128	DEL 49.3													T 50.5																											
Line #43-2	407 128	0													T 92.6						G 44.3																							

SlDET1 ON/OFF Target

			-30	-29	-28	-27	-26	-25	-24	-23	-22	-21	-20	-19	-18	-17	-16	-15	-14	-13	-12	-11	-10	-9	-8	-7	-6	-5	-4	-3	-2	-1	1	2	3	4	5	6	7	8	9	10	11	12
ON Target (*SlDET1*)	Sequence		A	C	T	A	T	C	A	G	G	A	C	C	T	G	A	A	G	C	T	G	G	C	A	G	C	A	C	A	G	A	T	G	G	G	G	A	A	C	T	A	A	G
Solyc01g056340: CDS	reads	Indel																																								
WT	351 660	0																																								
Line #16	265 118	0										T 69.2	T 57.5						T 49.6				T 49.3																			
													G 13.5																													
Line #16-1	216 182	0										T 66.5	T 61.8						T 49.0				T 50.1																			
													G 7.1																													
Line #16-2	183 108	0										T 60.8	T 68.1						T 49.8				T 48.6																			
Line #16-3	262 928	0										T 72.0	T 61.2						T 55.1				T 43.2																			
													G 11.5																													
Line #18	193 050	0										T 99.9	T 99.9																													
Line #43-1	475 814	0										T 62.9	T 62.9						T 48.3				T 51.5																			
Line #43-2	282 966	0										T 94.0	T 94.0						T 49.7				T 50.2																			

SlCYC ON/OFF Target

			-30	-29	-28	-27	-26	-25	-24	-23	-22	-21	-20	-19	-18	-17	-16	-15	-14	-13	-12	-11	-10	-9	-8	-7	-6	-5	-4	-3	-2	-1	1	2	3	4	5	6	7	8	9	10	11	12
ON Target (*SlCYC*)	Sequence		A	T	A	A	T	T	A	T	T	T	G	G	C	C	A	C	A	T	G	G	A	G	A	G	T	G	G	T	G	A	A	G	G	G	T	C	A	A	C	A	C	A
Solyc06g074240: CDS	reads	Indel																																								
WT	263 650	0																																								
Line #16	146 960	0													T 99.8	T 99.8		T 99.9																								
Line #16-1	246 338	INS 28													T 40.2	T 12.1		T 12.1																								
		DEL 87.7																																								
Line #16-2	217 104	DEL 16.7													T 83.2	T 83.2		T 68.1																								
Line #16-3	269 262	DEL 14													T 86.0	T 86.0		T 85.9																								
Line #18	283 062	0													T 99.7	T 99.7												A 11.2																
Line #43-1	267 398	0													T 99.9	T 99.9		T 99.9																								
Line #43-2	232 092	0													T 99.8	T 99.8		T 99.5																								

(b)

SNV position	-30	-29	-28	-27	-26	-25	-24	-23	-22	-21	-20	-19	-18	-17	-16	-15	-14	-13	-12	-11	-10	-9	-8	-7	-6	-5	-4	-3	-2	-1	1	2	3	4	5	6	7	8	9	10	11	12
SlDDB1 OFF Target2 Sequence	T	T	C	A	T	T	C	T	T	A	G	G	A	C	T	T	T	T	C	T	A	G	G	T	A	T	G	A	T	A	G	G	G	A	C	T	C	G	A	A	C	T
SlDET1 OFF Target1 Sequence	T	C	C	C	A	C	C	G	G	T	A	G	A	C	A	C	G	A	A	G	G	T	T	G	A	A	G	A	C	A	T	G	G	A	T	A	C	G	A	T	T	T
SlCYC OFF Target1 Sequence	A	G	A	G	A	G	G	G	C	A	A	G	C	G	A	G	A	T	T	G	A	G	A	G	T	G	G	T	G	A	G	G	G	A	G	A	G	A	G	G	G	C

SlDDB1 ON/OFF Target

OFF Target2 / Chr12: Intergenic	reads	Indel
WT	328 336	0
Line#16	52 390	0
Line#16-1	283 376	0
Line#16-2	283 376	0
Line#16-3	328 022	0
Line#18	294 646	0
Line#43-1	277 370	0
Line#43-2	286 202	0

SlDET1 ON/OFF Target

OFF Target1 / Solyc04g024750: Intergenic	reads	Indel
WT	336 046	0
Line#16	372 566	0
Line#16-1	307 084	0
Line#16-2	268 604	0
Line#16-3	292 522	0
Line#18	533 352	0
Line#43-1	254 300	0
Line#43-2	276 654	0

SlCYC ON/OFF Target

OFF Target1 / Ch03: Intergenic	reads	Indel
WT	245 666	0
Line#16	226 622	0
Line#16-1	186 370	0
Line#16-2	169 790	0
Line#16-3	173 078	0
Line#18	310 940	0
Line#43-1	230 538	0
Line#43-2	199 916	0

Frequency regarding the number of reads containing the nucleic acid

mutation frequency (%): 100, 40, 20, 10, 5, 1

DDB1, *DET1* and *CYC-B* on (a)- and off-target (b) mutation frequencies induced by Target-AID in tomato. The target sequence is highlighted in grey, and the mismatched bases of the off-target sites are in light blue. Mutation frequencies above 0.1% are shown and

highlighted as indicated at the bottom right. The total indel frequency among 42 bases (20 bases of the target region plus 10 upstream bases and 12 downstream bases) and SNV frequencies above 0.1% at each nucleotide position are shown and highlighted.

Supplementary Table S2 DNA modification spectra in triple-targeted T_0 plants.

Target	Line No.	PAM Target<br>5'-**CCATATCATACCTAGACAAT--------------GCT**TTTG-3'	Pattern	No. of reads with pattern / Total reads	No. of reads with DNA modifications /Total reads
DDB1 Target-AID site	#8	5'-CCATATCAT------------------------------TG-3'	-16	1/1	1/1
		5'-CCATATCATACCTAGACAAT--------------**a**CTTTTG-3'	S1		
	#16	5'-CCATATCATACCTAGACAAT--------------**a**CTTTTG-3'	S1	1/1	1/1
		5'-CCATATCATACCTAGACAAT---------------CTTTTG-3'	-1		
	#16-1	5'-CCATATCAT------------------------------TG-3'	-16	1/1	1/1
		5'-CCATATCATACCTAGACAAT--------------**a**CTTTTG-3'	S1		
	#16-2	5'-CCATATCATACCTAGACAAT--------------**a**CTTTTG-3'	S1	1/1	1/1
		5'-CCATATCAT---------------------------------3'	-25		
	#16-3	5'-CCATATCATACCTAGACAAT--------------**a**CTTTTG-3'	S1	8/9	9/9
		5'-CCATATCAT---------------------------------3'	-25	1/9	
	#18	5'-CCATATCATACCTAGACAAT**catacctagacaat**GCTTTTG-3'	+14	1/11	10/11
		5'-CCATA------------------------------CTTTTG-3'	-16	1/11	
		5'-CCATATCATACCTAGACAAT--------------**a**CTTTTG-3'	S1	8/11	
		5'-CCATATCATACCTAGACAAT--------------GCTTTTG-3'	WT	1/11	
	#26	5'-CCATATCATACCTAGACAAT--------------GCTTTTG-3'	WT	1/1	1/1
		5'-CCATATCATACCTAGACAAT--------------**a**CTTTTG-3'	S1		
		5'-CCATATCATTACCTA-------------------**a**CTTTTG-3'	-6, S2		
	#27	5'-CCATATCATACCTA**c**ACAAT--------------**a**CTTTTG-3'	S2	1/1	1/1
	#30	5'-CCATATCATACCTAGACAAT--------------GCTTTTG-3'	WT	1/1	1/1
		5'-CCATATCATACCTA**c**ACAAT--------------**a**CTTTTG-3'	S2	1/1	1/1
	#36-2	5'-CCATATCATACCTAGACAAT--------------GCTTTTG-3'	WT	1/1	1/1

	#43-1	5'-CCATATCATACCTAGACAAT---------------CTTTTG-3'	-1	1/1	1/1
		5'-CCATATCATACCTAcACAAT--------------aCTTTTG-3'	S2		
	#43-2	5'-CCATATCATACCTAcACAAT--------------aCTTTTG-3'	S2	4/9	8/9
		5'-CCATATCATACCTAGACAAT--------------aCTTTTG-3'	S1	4/9	
		5'-CCATATCATACCTAGACAAT--------------GCTTTTG-3'	WT	1/9	
Target	Line No.	Target PAM<br>5'-AGG**ACCTGAAGCTGG-CAGCACAGA****TGG**-3'	Pattern	No. of reads with pattern / Total reads	No. of reads with DNA modifications /Total reads
DET1 Target-AID site	#8	5'-AGGAttTGAAGCTGG-CAGCACAGATGG-3'	S2	1/1	1/1
	#16	5'-AGGAttTGAAGtTGG-tAGCACAGATGG-3'	S4	1/1	1/1
		5'-AGGAtgTGAAGtTGG-tAGCACAGATGG-3'	S4		
		5'-AGGACCTGAAGCTGG-CAGCACAGATGG-3'	WT		
	#16-1	5'-AGGAttTGAAGtTGG-tAGCACAGATGG-3'	S4	1/1	1/1
		5'-AGGACCTGAAGCTGG-CAGCACAGATGG-3'	WT		
	#16-2	5'-AGGAttTGAAGtTGG-tAGCACAGATGG-3'	S4	1/1	1/1
		5'-AGGACCTGAAGCTGG-CAGCACAGATGG-3'	WT		
	#16-3	5'-AGGAttTGAAGtTGG-CAGCACAGATGG-3'	S3	3/7	3/7
		5'-AGGACCTGAAGCTGG-CAGCACAGATGG-3'	WT	4/7	
	#18	5'-AGGAttTGAAGtTGG-CAGCACAGATGG-3'	S3	12/15	14/15
		5'-AGGAttTGAAGCTGG-CAGCACAGATGG-3'	S2	12/15	
		5'-AGGACCTGAAGCTGG-CAGCACAGATGG-3'	WT	1/15	
	#26	5'-AGGAttTGAAGCTGG-tAGCACAGATGG-3'	S3	1/1	1/1
		5'-AGGACCTGAAGCTGGaCAGCACAGATGG-3'	+1		
		5'-AGGACCTGAAGCTGG-CAGCACAGATGG-3'	WT		
	#27	5'-AGGAttTGAAGCTGG-CAGCACAGATGG-3'	S2	1/1	1/1

		5'-AGGACCTGAAGCTGG-CAGCACAGATGG-3'	WT		
	#30	5'-AGGAttTGAAGCTGG-tAGCACAGATGG-3'	S3	1/1	1/1
		5'-AGGA---------GG-CAGCACAGATGG-3'	-9		
		5'-AGGACCTGAAGCTGG-CAGCACAGATGG-3'	WT		
	#36-2	5'-AGGACtTGAAGCTGG-CAGCACAGATGG-3'	S1	1/1	1/1
		5'-AGGACCTGAAGCTGG-CAGCACAGATGG-3'	WT		
	#43-1	5'-AGGAttTGAAGtTGG-tAGCACAGATGG-3'	S4	1/1	1/1
		5'-AGGACCTGAAGCTGG-CAGCACAGATGG-3'	WT		
	#42-2	5'-AGGAttTGAAGtTGG-CAGCACAGATGG-3'	S3	4/4	4/4
Target	Line No.	**PAM** Target<br>5'-**CCT**TCACCACTCTCCATGTGGCCAAAT-3'	Pattern	No. of reads with pattern / Total reads	No. of reads with DNA modifications /Total reads
CYC-B Target-AID site	#8	5'-CCTTCACCACTCTCCATGTaaCCAAAT-3'	S2	1/1	1/1
	#16	5'-CCTTCACCACTCTCCATaTaaCCAAAT-3'	S3	1/1	1/1
	#16-1	5'-CCTTCAC-------C-----GCCAAAT-3'	-12	1/1	1/1
		5'-CCTTCACCACTCTCCATaTaaCCAAAT-3'	S3		
	#16-2	5'-CCTTCACCACTCTCCATaTaaCCAAAT-3'	S3	1/1	1/1
	#16-3	5'-CCTTCAC-------C-----GCCAAAT-3'	-12	1/6	6/6
		5'-CCTTCACCACTCTCCATaTaaCCAAAT-3'	S3	5/6	
	#18	5'-CCTTCACCACTCTCCATGTaaCCAAAT-3'	S2	8/8	8/8
	#26	5'-CCTTCACCACTCTCCATGTaaCCAAAT-3'	S2	1/1	1/1
		5'-CCTTCACCACTCTCCATaTaaCCAAAT-3'	S3		
	#27	5'-CCTTCACCACTCTCCATaTaaCCAAAT-3'	S3	1/1	1/1
		5'---------ACTCTCCATGTGGCCAAAT-3'	-41		
	#30	5'-CCTTCACCACTCTCCATaTaaCCAAAT-3'	S3	1/1	1/1

		5'-CCTTCACCACTCTCCATGTGGCCAAAT-3'	WT		
		ND	INDEL		
	#36-2	5'-CCTTCACCACTCTCCATGTGGCCAAAT-3'	WT	1/1	1/1
	#43-1	5'-CCTTCACCACTCTCCAT**a**T**aa**CCAAAT-3'	S3	1/1	1/1
	#43-2	5'-CCTTCACCACTCTCCAT**a**T**aa**CCAAAT-3'	S3	10/10	10/10

No. of plants: the number of T_0 plants having the combination of each DNA modification pattern.

Rate of mutated plants: the number of genome-editing T_0 plants per total examined plants.

ND: non determined

Yellow cells correspond to sequence containing substitution

Green cells correspond to direct PCR product sequence

Supplementary Table S3 Segregation patterns of mutations in triple-targeted T_1 plants.

Target	Line No.	No. of examined plants	PAM Target<br>5'-**CCA**TATCAT---ACCTA--GACAATGCTTTTG-3'	Pattern	Zygous	No. of plants	Rate of mutated plants
DDB1 Target-AID site	#16	10	5'-CCATATCAT---ACCTA--GACAATaCTTTTG-3'	S1	Homo	7	10/10
			5'-CCATATCAT---ACCTA--cACAATaCTTTTG-3'	S2	Homo	1	
			5'-CCATATCAT---ACCTA--GACAATaCTTTTG-3'	S1	Biallelic	1	
			5'-CCATATCAT---ACCTA--GACAAT-CTTTTG-3'	-1			
			5'-CCATATCAT---ACCTA--cACAATaCTTTTG-3'	S2	Biallelic	1	
			5'-CCATATCAT---ACCTA--GACAATaCTTTTG-3'	S1			
	#16-1	3	5'-CCATATCAT---ACCTA--GACAATGCTTTTG-3'	WT	-	1	3/3
			5'-CCATATCAT---ACCTA--GACAATGCTTTTG-3'	WT	Hetero	1	
			5'-CCATATCAT---ACCTA--GACAATaCTTTTG-3'	S1			
			5'-CCATATCAT---ACCTA--cACAATaCTTTTG-3'	S2	Biallelic	3	
			5'-CCATATCAT---ACCTA--GACAATaCTTTTG-3'	S1			
	#16-2	11	5'-CCATATCAT---ACCTA--GACAATaCTTTTG-3'	S1		5	11/11
			5'-CCATATCAT---ACCTA--cACAATaCTTTTG-3'	S2	Biallelic	5	
			5'-CCATATCAT---ACCTA--GACAATaCTTTTG-3'	S1			
			5'-CCATATCAT---ACCTA--cACAATaCTTTTG-3'	S2	Biallelic	1	
			5'-CCATATCAT---ACCTA--G--AATaCTTTTG-3'	S1, -2			
	#16-3	8	5'-CCATATCAT---ACCTA--GACAATaCTTTTG-3'	S1	Homo	6	8/8
			5'-CCATATCAT---ACCTA--GACAATaCTTTTG-3'	S1	Chimeric	2	
			5'-CCATATCAT---ACCTA--cACAATaCTTTTG-3'	S2			
			5'-CCATATCAT---ACCTA--aACAATaCTTTTG-3'	S2			

	#18	24	5'-CCATATCATcatACCTA--GACAATGCTTTTG-3'	+3	Hetero	1	12/24
			5'-CCATATCAT---ACCTA--GACAATGCTTTTG-3'	WT			
			5'-CCATATCAT---ACCTA--GACAATaCTTTTG-3'	S1	Biallelic	4	
			5'-CCATATCATcatACCTA--GACAATGCTTTTG-3'	+3			
			5'-CCATATCAT---ACCTA--GACAATaCTTTTG-3'	S1	Homo	6	
			5'-CCATATCAT---ACCTA--GACAATcCTTTTG-3'	S1	Biallelic	1	
			5'-CCATATCAT---ACCTA--GACAATaCTTTTG-3'	S1			
			5'-CCATATCAT---ACCTA--GACAATGCTTTTG-3'	WT	-	12	
	#43-1	6	5'-CCATATCAT---ACCTA--tACAATaCTTTTG-3'	S2	Biallelic	1	6/6
			5'-CCATATCAT---ACCTA--cACAATaCTTTTG-3'	S2			
			5'-CCATATCAT---ACCTA--GACAATaCTTTTG-3'	S1	Homo	3	
			5'-CCATATCAT---ACCTA--cACAATaCTTTTG-3'	S2	Biallelic	2	
			5'-CCATATCAT---ACCTA--GACAATaCTTTTG-3'	S1			
	#43-2	11	5'-CCATATCAT---ACCTA--cACAATaCTTTTG-3'	S2	Biallelic	5	11/11
			5'-CCATATCAT---ACCTA--GACAATaCTTTTG-3'	S1			
			5'-CCATATCAT---ACCTA--aACAATaCTTTTG-3'	S2	Biallelic	2	
			5'-CCATATCAT---ACCTA--GACAATaCTTTTG-3'	S1			
			5'-CCATATCAT---ACCTAtaGACAATaCTTTTG-3'	S1, +2	Biallelic	1	
			5'-CCATATCAT---ACCTA--GACAATaCTTTTG-3'	S1			
			5'-CCATATCAT---ACCTA--GACAATaCTTTTG-3'	S1	Homo	3	
Target	Line No.	No. of examined plants	Target PAM<br>5'-AGGACCTGAAGCTGGCAGCACAGATGG-3'	Pattern	Zygous	No. of plants	Rate of mutated plants
	#16	10	5'-AGGAttTGAAGtTGGCAGCACAGATGG-3'	S3	Homo	2	10/10
			5'-AGGAttTGAAGtTGGtAGCACAGATGG-3'	S4	Hetero	3	

DET1 Target-AID site			5'-AGGACCTGAAGCTGGCAGCACAGATGG-3'	WT			
			5'-AGGAttTGAAGCTGGCAGCACAGATGG-3'	S2	Biallelic	1	
			5'-AGGAttTGAAGtTGGCAGCACAGATGG-3'	S3			
			5'-AGGAttTaAAGtTGGCAGCACAGATGG-3'	S4	Biallelic	1	
			5'-AGGAttTGAAGtTGGCAGCACAGATGG-3'	S3			
			5'-AGGAttTaAAGtTGGCAGCACAGATGG-3'	S4	Biallelic	2	
			5'-AGGACCTGAAGCTGGCAGCACAGATGG-3'	WT			
			ND	ND	Biallelic	1	
			5'-AGGAttTGAAGtTGGCAGCACAGATGG-3'	S3			
	#16-1	3	5'-AGGAttTGAAGtTGGtAGCACAGATGG-3'	S4	Homo	2	3/3
			5'-AGGAttTGAAGtT---AGtACAGATGG-3'	S4, -3	Hetero	1	
			5'-AGGACCTGAAGCTGGCAGCACAGATGG-3'	WT			
	#16-2	11	5'-AGGAttTGAAGtTGGtAGCACAGATGG-3'	S4	Hetero	2	11/11
			5'-AGGACCTGAAGCTGGCAGCACAGATGG-3'	WT			
			5'-AGGAttTGAAGtTGGtAGCACAGATGG-3'	S4	Biallelic	4	
			5'-AGGAttTGAAGCTGGCAGCACAGATGG-3'	S2			
			5'-AGGAttTGAAGtTGGtAGCACAGATGG-3'	S4	Hetero	2	
			5'-AGGACtTGAAGCTGGCAGCACAGATGG-3'	S1			
			5'-AGGAttTGAAGtTGGtAGCACAGATGG-3'	S4	Biallelic	1	
			5'-AGGACtTGAAGCTGGtAGCACAGATGG-3'	S2			
			5'-AGGAttTGAAGCTGGtAGCACAGATGG-3'	S3	Chimeric	1	
			5'-AGGAtgTGAAGCTGGtAGCACAGATGG-3'	S3			
			5'-AGGACCTGAAGCTGGtAGCACAGATGG-3'	S1			
			5'-AGGACCTGAAGCTGGtAGCACAGATGG-3'	S1	Homo	1	

	#16-3	8	5'-AGGAttTGAAGtTGGCAGCACAGATGG-3'	S3	Homo	8	8/8
	#18	24	5'-AGGAttTGAAGCTGGCAGCACAGATGG-3'	S2	Homo	11	18/24
			5'-AGGAttTGAAGCTGGCAGCACAGATGG-3'	S2	Biallelic		
			5'-AGGAttTGAAGcTGGCAGCACAGATGG-3'	S3			
			5'-AGGAttTGAAGCTGGCAGCACAGATGG-3'	S2	Hetero		
			5'-AGGACCTGAAGCTGGCAGCACAGATGG-3'	WT			
			5'-AGGAttTGAAGtTGGCAGCACAGATGG-3'	S3	Hetero		
			5'-AGGACCTGAAGCTGGCAGCACAGATGG-3'	WT			
			5'-AGGACCTGAAGCTGGCAGCACAGATGG-3'	WT	-	6	
	#43-1	6	5'-AGGAttTGAAGtTGGCAGCACAGATGG-3'	S3	Homo	1	6/6
			5'-AGGAttTGAAGtTGGCAGCACAGATGG-3'	S3	Biallelic	2	
			5'-AGGAtgTGAAGtTGGCAGCACAGATGG-3'	S3			
			5'-AGGAttTGAAGtTGGaAGCACAGATGG-3'	S4	Chimeric	1	
			5'-AGGAttTGAAGtTGGtAGCACAGATGG-3'	S4			
			5'-AGGACCTGAAGCTGGCAGCACAGATGG-3'	WT			
			5'-AGGAttTGAAGtTGGtAGCACAGATGG-3'	S4	Hetero	2	
			5'-AGGACCTGAAGCTGGCAGCACAGATGG-3'	WT			
	#42-2	11	5'-AGGAttTGAAGtTGGCAGCACAGATGG-3'	S3	Biallelic	3	11/11
			5'-AGGAttTGAAGCTGGCAGCACAGATGG-3'	S2			
			5'-AGGAttTGAAGtTGGtAGCACAGATGG-3'	S4	Biallelic	4	
			5'-AGGAttTGAAGCTGGCAGCACAGATGG-3'	S2			
			5'-AGGAttTGAAGCTGGtAGCACAGATGG-3'	S3	Homo	4	
Target	Line No.	No. of examined plants	PAM Target<br>5'-CCTTCACCACTCTCCATGTGGCCAAAT-3'	Pattern	Zygous	No. of plants	Rate of mutated plants

CYC-B Target-AID site	#16	10	5'-CCTTCACCACTCTCCATaTaaCCAAAT-3'	S3	Homo	6	10/10
			5'-CCTTCACCACTCTCCATaTaaCCAAAT-3'	S3	Hetero	4	
			5'-CCTTCACCACTCTCCATGTGGCCAAAT-3'	WT			
	#16-1	3	5'-CCTTCACCAC---------GaCCAAAT-3'	S1, -9	Homo	2	3/3
			5'-CCTTCACCAC---------GaCCAAAT-3'	S1, -9	Biallelic	1	
			5'-CCTTCAC------------GaCCAAAT-3'	S1, -12			
	#16-2	11	5'-CCTTCACCACTCTCCATaTaaCCAAAT-3'	S3	Hetero	1	11/11
			5'-CCTTCACCACTCTCCATGTGGCCAAAT-3'	WT			
			5'-CCTTCACCACTCTCCATaTaaCCAAAT-3'	S3	Homo	10	
	#16-3	8	5'-CCTTCACCACTCTCCATaTaaCCAAAT-3'	S3	Homo	8	8/8
	#18	24	5'-CCTTCACCACTCTCCATGTaaCCAAAT-3'	S2	Homo	13	13/24
			5'-CCTTCACCACTCTCCATGTGGCCAAAT-3'	WT	-	11	
	#43-1	6	5'-CCTTCACCACTCTCCATaTaaCCAAAT-3'	S3	Homo	1	6/6
			5'-CCTTCACCACTCTCCATGTGGCCAAAT-3'	WT			
			5'-CCTTCACCACTCTCCATaTaaCCAAAT-3'	S3	Homo	5	
	#43-2	11	5'-CCTTCACCACTCTCCATaTaaCCAAAT-3'	S3	Hetero	3	10/11
			5'-CCTTCACCACTCTCCATGTGGCCAAAT-3'	WT			
			5'-CCTTCACCACTCTCCATaTaaCCAAAT-3'	S3	Biallelic	1	
			ND	ND			
			5'-CCTTCACCACTCTCCATaTaaCCAAAT-3'	S3	Homo	7	

No. of plants: the number of T_1 plants having the combination of each DNA modification pattern.

Rate of mutated plants: the number of genome-editing T_1 plants per total examined plants.

Yellow cells correspond to sequence containing substitution

Supplementary Table S4. Marker gene-free *SlDDB1-SlDET1-SlCYC-B*-targeted transgenic plants with stably inherited DNA mutations

Line [#T_0 no.]	Segregation null/total	Line [#T_0 no._offspring no.]	Primary null-segregant		
			Zygosity *DDB1*	Zygosity *DET1*	Zygosity *CYC-B*
#16	2/10	#16_3	S2/S2 [Homo]	S4/WT [Hetero]	S3/WT [Hetero]
		#16_7	S1/S1 [Homo]	S4/WT [Hetero]	S3/S3 [Homo]
#16-1	1/3	#16-1_1	WT/WT [Homo]	WT/WT [Homo]	(-9 and S1)(/-12 and S1) [Biallelic]
#16-2	2/11	#16-2_2	S1/S1 [Homo]	S4/WT [Hetero]	S3/S3 [Homo]
		#16-2_3	S1/S1 [Homo]	S3/S3 [Homo]	S3/S3 [Homo]
#16-3	1/8	#16-3_3	S1/S1 [Homo]	S3/S3 [Homo]	S3/S3 [Homo]
#18	12/24	#18_3	WT/WT [Homo]	WT/WT [Homo]	WT/WT [Homo]
		#18_4	WT/WT [Homo]	WT/WT [Homo]	WT/WT [Homo]
		#18_6	WT/WT [Homo]	WT/WT [Homo]	WT/WT [Homo]
		#18_7	WT/WT [Homo]	WT/WT [Homo]	WT/WT [Homo]
		#18_11	WT/WT [Homo]	WT/WT [Homo]	WT/WT [Homo]
		#18_2'	WT/WT [Homo]	S2/WT [Hetero]	WT/WT [Homo]
		#18_4'	WT/WT [Homo]	WT/WT [Homo]	WT/WT [Homo]
		#18_7'	WT/WT [Homo]	S3/WT [Hetero]	WT/WT [Homo]
		#18_8'	WT/WT [Homo]	S3/WT [Hetero]	WT/WT [Homo]
		#18_9'	WT/WT [Homo]	S1/WT [Hetero]	S2/S2 [Homo]
		#18_11'	WT/WT [Homo]	S3/WT [Hetero]	WT/WT [Homo]
		#18_12'	WT/WT [Homo]	S3/WT [Hetero]	WT/WT [Homo]
#43-2	4/11	#43-2_2	S2/S1 [Biallelic]	S2/S2 [Homo]	S3/S3 [Homo]
		#43-2_1'	S1/S1 [Homo]	S2/S4 [Biallelic]	S3/WT [Hetero]
		#43-2_6'	S1/S1 [Homo]	S2/S3 [Biallelic]	S3/S3 [Homo]
		#43-2_7'	S1/S1 [Homo]	S3/S3 [Homo]	S3/S3 [Homo]

Line, # indicates parental line, and following numbers indicate individual sibling plants.

Segregation null/total: the first generation that carried no transgene on total number of T1 analysed.

Primary null-segregant: the first generation that carried no transgene.

Zygosity: type of stably inherited mutation.

The mutation spectrum in marker-gene free plants that correspond to those examined in (a). Primary null-segregant, the first generation that carried no transgene; Zygosity, type of stably inherited mutation.

Supplementary Table S5. Primers used in this study

Name	Sequence	Amplified region	Used for
NPTII-F	5'-ATGATTGAACAAGATGGATTGCAC-3'	*NPTII*	Detection of transgene
NPTII-R	5'-TCAGAAGAACTCGTCAAGAAGGCG-3'	*NPTII*	Detection of transgene
Actin-F	5'-GATGGATCCTCCAATCCAGACACTGTA'-3'	*Actin*	Detection of genome DNA
Actin-R	5'-GTATTGTGTTGGACTCTGGTGATGGTGT'-3'	*Actin*	Detection of genome DNA
DDB1_ON target-F1	5'-GCATATGGGCGGGTTGATGCTGATG'-3'	*SlDDB1*	Cloning and Sequence
DDB1_ON target-R1	5'-GCCTTTCCAGATCAACAACACAGAAGTCCA-3'	*SlDDB1*	Cloning
DET1_ON target-F1	5'-TCAGCCCTCTCATCCATACC-3'	*SlDET1*	Cloning and Sequence
DET1_ON target-R1	5'-CGTCTTGGCACTCTATCAAGC-3'	*SlDET1*	Cloning
CYC_ON target-F1	5'-TCCACCTCCCTCCATAATTA-3'	*SlCYC*	Cloning and Sequence
CYC_ON target-R1	5'-CACTCTGTTCTCAACACAAC-3'	*SlCYC*	Cloning
SlDDB1_1st_F	GCTTCTTTGCTAATACCAGTACCTCCACCA	*SlDDB1*	Target Sequence
SlDDB1_1st_R	TCCTTTGATCCCTTGTAGTTCCACAGACGC	*SlDDB1*	Target Sequence
DDB1_OFF target_1st_F	CGTGAACAACTCTAGAATCCAAG	*SlDDB1_OFF_target*	Target Sequence
DDB1_OFF target_1st_R	ACGAACCCGTTGTCTAGAGTCA	*SlDDB1_OFF_target*	Target Sequence
SlDDB1_2nd_F	tctttccctacacgacgctcttccgatctGCTGGGAATGAAATCTTCTCTTG	*SlDDB1*	Target Sequence
SlDDB1_2nd_R	gtgactggagttcagacgtgtgctcttccgatctGCAACTGAAATTTAAAACCTCAATG	*SlDDB1*	Target Sequence
DDB1_OFF_Target_2nd_F	tctttccctacacgacgctcttccgatctCGAAAGTCATACTTAAGTAAATAAAGAAGA	*SlDDB1_OFF_target*	Target Sequence
DDB1_OFF_Target_2nd_R	gtgactggagttcagacgtgtgctcttccgatctGCTTTTATGGTCTATGTCAGTTATTGC	*SlDDB1_OFF_target*	Target Sequence
SlDET1_1st_F	GCAAGTTGATAGGAGGGTATTTTTGTTTCT	*SlDET1*	Target Sequence
SlDET1_1st_R	CATACTAACCGTCTTGGCACTCTATCAAGC	*SlDET1*	Target Sequence
DET1_OFF target_F	GTAAACCTAAGGCGCATAGCACC	*SlDDB1_OFF_target*	Target Sequence
DET1_OFF target_R	TCCCCAAGGATAATAAATAAGGCG	*SlDDB1_OFF_target*	Target Sequence

SlDET1_2nd_F	tctttccctacacgacgctcttccgatctGCCATAGACAGTCTACTGACCATCC	*SlDET1*	Target Sequence
SlDET1_2nd_R	gtgactggagttcagacgtgtgctcttccgatctGATATTTACAACTGATGCCTGCAG	*SlDET1*	Target Sequence
DET1_OFF-target_2nd_F	tctttccctacacgacgctcttccgatctGCTAGCACAGGAAGCTCGAA	*SlDET1_OFF_target*	Target Sequence
DET1_OFF-target_2nd_R	gtgactggagttcagacgtgtgctcttccgatctGCTGATCCTGCTACATCTGAGC	*SlDET1_OFF_target*	Target Sequence
SlCYC1_1st_F	GTTCCACCTCCCTCCATAATTAACCATTAT	*SlCYC*	Target Sequence
SlCYC1_1st_R	CGATAAAACAGGACGACTCACCAAAGAAG	*SlCYC*	Target Sequence
CYC1_Off1_1st_F	GCCATGTCACTGCATGTGTATCAC	*SlCYC_OFF_target*	Target Sequence
CYC1_Off1_1st_R	ATTACCTTTCATGTGAAGCCCATCG	*SlCYC_OFF_target*	Target Sequence
SlCYC1_2nd_F	tctttccctacacgacgctcttccgatctGCCAGAGTCTTTAGATGTTAAC	*SlCYC*	Target Sequence
SlCYC1_2nd_R	gtgactggagttcagacgtgtgctcttccgatctGCTTCTTTCTACTAACTCTACCATA	*SlCYC*	Target Sequence
CYC1_OFF-target_2nd_F2	tctttccctacacgacgctcttccgatctCGTGGGGTGACTTCGAATTT	*SlDET1_OFF_target*	Target Sequence
CYC1_OFF-target_2nd_R2	gtgactggagttcagacgtgtgctcttccgatctCGAGACTGGGAGAGAGGTAAGC	*SlDET1_OFF_target*	Target Sequence
AtU6_F	5'-CTTTGTACAAAAAAGCAGGCG-3'	U6 promoter	target vector
AtU6_R	5'-GCCATAGAAAAGTTGGGTG-3'	U6 promoter	target vector
gRNA_R	5'-CAATCACTACTTCGACTCTAG-3'	gRNA	target vector
SlDDB1_target1	5'-ctagagtcgaagtagtgattgAGCATTGTCTAGGTATGATAgttttagagctagaaatagcaag-3'	*SlDDB1*	target vector (for SlDDB1)
SlDET1_target2	5'-ctagagtcgaagtagtgattgACCTGAAGCTGGCAGCACAGAgttttagagctagaaatagcaag-3'	*SlDET1*	target vector (for SlDET1)
SlCYC-B_target3	5'-ctagagtcgaagtagtgattgGGCCACATGGAGAGTGGTGAgttttagagctagaaatagcaag-3'	*SlCYC-B*	target vector (for SlCYC-B)
kn2052_UGI_F	5'-CTAAGAAGAAACGTAAAGTAgggcCcATGACCAACCTTTCCGAC-3'	*UGI* insertion	Vector construction for pDicAID_nCAS9-PmCDA_UGI_NptII
kn2053_UGI_R	5'-agctgggaggcctggatcAgGgctatgcaaccagtccTAGCATC-3'		
